# A Dynamic Stress Model Explains the Delayed Drug Effect in Artemisinin Treatment of Plasmodium falciparum

**DOI:** 10.1128/AAC.00618-17

**Published:** 2017-11-22

**Authors:** Pengxing Cao, Nectarios Klonis, Sophie Zaloumis, Con Dogovski, Stanley C. Xie, Sompob Saralamba, Lisa J. White, Freya J. I. Fowkes, Leann Tilley, Julie A. Simpson, James M. McCaw

**Affiliations:** a School of Mathematics and Statistics, The University of Melbourne, Melbourne, Australia; b Department of Biochemistry and Molecular Biology and Australian Research Council Centre of Excellence for Coherent X-Ray Science, Bio21 Molecular Science and Biotechnology Institute, The University of Melbourne, Melbourne, Australia; c Centre for Epidemiology and Biostatistics, Melbourne School of Population and Global Health, The University of Melbourne, Melbourne, Australia; d Mahidol-Oxford Tropical Medicine Research Unit, Faculty of Tropical Medicine, Mahidol University, Rajthevee, Bangkok, Thailand; e Burnet Institute, Melbourne, Australia; f Modelling and Simulation, Infection and Immunity Theme, Murdoch Childrens Research Institute, The Royal Children's Hospital, Parkville, Victoria, Australia

**Keywords:** Plasmodium falciparum, artemisinin action, drug exposure time, dynamic model

## Abstract

Artemisinin resistance constitutes a major threat to the continued success of control programs for malaria, particularly in light of developing resistance to partner drugs. Improving our understanding of how artemisinin-based drugs act and how resistance manifests is essential for the optimization of dosing regimens and the development of strategies to prolong the life span of current first-line treatment options. Recent short-drug-pulse *in vitro* experiments have shown that the parasite killing rate depends not only on drug concentration but also the exposure time, challenging the standard pharmacokinetic-pharmacodynamic (PK-PD) paradigm in which the killing rate depends only on drug concentration. Here, we introduce a dynamic stress model of parasite killing and show through application to 3D7 laboratory strain viability data that the inclusion of a time-dependent parasite stress response dramatically improves the model's explanatory power compared to that of a traditional PK-PD model. Our model demonstrates that the previously reported hypersensitivity of early-ring-stage parasites of the 3D7 strain to dihydroartemisinin compared to other parasite stages is due primarily to a faster development of stress rather than a higher maximum achievable killing rate. We also perform *in vivo* simulations using the dynamic stress model and demonstrate that the complex temporal features of artemisinin action observed *in vitro* have a significant impact on predictions for *in vivo* parasite clearance. Given the important role that PK-PD models play in the design of clinical trials for the evaluation of alternative drug dosing regimens, our novel model will contribute to the further development and improvement of antimalarial therapies.

## INTRODUCTION

Plasmodium falciparum malaria is a major vector-borne parasitic disease affecting over 200 million people annually (1). Over the past 2 decades artemisinin-based therapies, used as the first-line treatment against falciparum malaria, have been shown to be highly effective. Their wide-scale distribution (approximately 390 million treatment courses delivered annually) has been instrumental in achieving a dramatic reduction in morbidity and mortality through both individual-level clinical and public health benefits (1). Worryingly, over the past decade P. falciparum parasites resistant to artemisinin derivatives, originally defined via a clinical phenotype of decreased parasite clearance rate following treatment and now characterized by the presence of the K13 mutation, have begun to emerge and spread across Southeast Asia (2, 3, 4, 5). With no new antimalarial drugs yet available and alternatives unlikely to be brought to market within the next few years, advancing our understanding of the antimalarial action of the artemisinins is essential to prolong the life span of the current first-line treatment for malaria.

A model-based study of clinical isolates from Pailin (western Cambodia) by Saralamba et al. demonstrated that artemisinin-resistant parasites displayed a reduced sensitivity to artesunate (an artemisinin derivative with the active metabolite dihydroartemisinin [DHA]) during the ring stage of infection (6). Recent *in vitro* experiments have further demonstrated that P. falciparum exhibits a distinct stage-dependent susceptibility to artemisinin and that resistant isolates show a reduced drug susceptibility during the very early ring stage of development (7, 8, 9). Despite this developing understanding of the subtleties of artemisinin action and drug resistance, a major gap remains in describing the full dynamics of the host-pathogen-drug system and translating findings from the well-controlled *in vitro* experimental environment to the *in vivo* context.

Pharmacokinetic-pharmacodynamic (PK-PD) modeling, which integrates drug kinetics (e.g., absorption and elimination) with the dynamics of both cyclic parasite growth and drug-parasite interactions, enables the quantitative assessment of drug efficacy, determination of optimal dosing schemes, and advancement of our understanding of antimalarial action and resistance (6, 10, 11, 12, 13, 14, 15, 16, 17, 18, 19). Over nearly 20 years of development, PK-PD models have increased significantly in complexity. Building from early models, which treated infected red blood cells as a single compartment (10, 11, 12), models have expanded to capture the different stages of the parasite life cycle in the red blood cell (ring, trophozoite, and schizont), allowing the incorporation of stage-dependent drug effects (6, 13, 19). A feature common to almost all PK-PD models of artemisinin-based therapy developed to date has been the implicit assumption that the relationship between drug concentration and the rate of parasite killing is independent of the history of exposure (an exception is a turnover model proposed by Patel et al. [16], which assumed an unidentified physiological process mediating the parasite killing). The transient killing rate, *k* (i.e., the fraction of parasites killed by drug per unit of time), has been empirically modeled by a Hill function of plasma drug concentration (*C*),

(1) $$k(C)=\frac{k_{\text{max}}C^{\gamma }}{{K_{c}}^{\gamma }+C^{\gamma }}$$

where parameters *k*_max_ (maximum killing rate), γ (Hill coefficient), and *K_c_* (half-maximal killing concentration) are (possibly stage-dependent) fixed quantities (i.e., constants) (20). The killing rate varies with drug concentration in a sigmoidal manner and saturates at the *k*_max_ for high drug concentration. Under this formulation, a higher (nonsaturating) drug concentration will immediately exert a stronger killing effect.

However, the recent *in vitro* experiments of Klonis et al. (7) and Dogovski et al. (9) have provided clear evidence that a higher (nonsaturating) drug concentration may not result in an increased rate of killing. Indeed, for sufficiently short exposure times, the cumulative killing effect (i.e., one minus the fraction of parasites surviving the exposure) may be strongly limited and largely independent of drug concentration. These observations are in direct contradiction with the possible behavior displayed by a model in which the killing rate is solely concentration dependent (equation 1). Klonis et al. and Dogovski et al. have demonstrated that the fraction of parasites that remains viable (i.e., able to asexually reproduce and initiate a subsequent round of blood-stage infection) does not depend solely on the applied drug concentration. Rather, viability was established to be a complex function of the drug exposure time and the initial drug concentration, manifesting as (stage-dependent) variations in the exposure time required to render parasites nonviable (7). The minimum exposure time required for loss of viability was particularly extended for mid-ring-stage parasites (artemisinin-sensitive 3D7 laboratory strain). Antimalarial resistance corresponded to a distinct change in the susceptibility of early-ring-stage parasites (9). To date, these novel properties have not been incorporated into a mechanistic model of parasite killing, indicating a requirement to extend the PK-PD modeling framework to reflect our emerging understanding of drug activity and evaluate the influence of these novel biological phenomena on the prediction of parasite clearance (20).

In this paper, we generalize the traditional model of killing (equation 1) by allowing the *k*_max_ and *K_c_* to be time-dependent quantities and then fit the generalized model to viability data for the 3D7 laboratory strain available in reference 7. By doing so, we aim to (i) show if the model is able to capture the full set of *in vitro* viability data (and also if the generalized model is statistically superior to the traditional model) and (ii) elucidate how the artemisinin-mediated killing effect develops following drug exposure and how that development differs between the parasite life stages. These results will further imply the relative contributions of drug concentration and exposure time to the effective killing rate. Finally, we incorporate the time-dependent drug effect into a PK-PD modeling framework to evaluate its effect on *in vivo* parasite killing. Complex temporal effects are anticipated to be present, as the short half-life of the artemisinins *in vivo* is comparable to the exposure time required for effective parasite killing.

## RESULTS

### Construction of the dynamic stress model.

In order to identify the key features that motivate the development of our model, we first review the *in vitro* experimental procedure (see reference 7 for details). Cultures containing equal quantities of tightly age-synchronized P. falciparum parasites (3D7 laboratory strain; over 80% of parasites synchronized within a 1-h age window) were treated with a specified dose of dihydroartemisinin (DHA) for a duration of 1, 2, 4, or 6 h before washing (to remove all drug). To quantify the effect of drug, a viability assay was performed. Viable parasites were defined as those able to reproduce and enter the next cycle of replication (thus excluding dead and dormant populations), assessed by measuring the parasitemia (*P*) in the trophozoite stage in the following life cycle 48 h later. In order to calculate the viability, parasitemia was also measured for two special cases: the control case (*P*_control_), where no drug was applied, and the background case (*P*_background_) with supermaximal DHA concentration (>10× the 50% lethal dose of 3 days, in nanomolars), applied for more than 48 h. Viability (*V*), a unitless ratio, then was given by subtracting the unviable population,

(2) $$V=\frac{P-P_{\text{background}}}{P_{\text{control}}-P_{\text{background}}}$$

**FIG 1:**
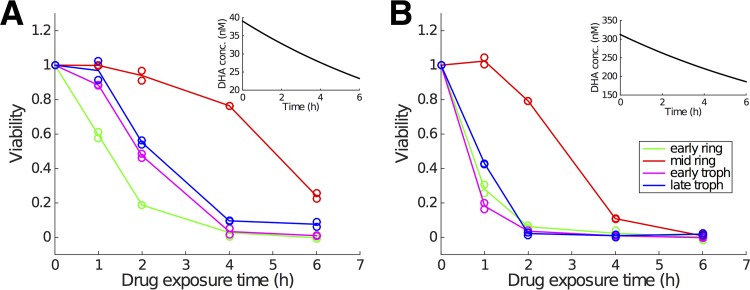
Representative experimental data showing how the fraction of viable parasite (i.e., viability) changes with the duration of drug exposure for two different initial DHA concentrations (39 nM [left] and 300 nM [right]) and four different parasite life stages. For one parasite stage and one drug exposure time in each panel, duplicate viability measurements are provided, which means in total 40 data points are measured in each panel (note that viability for zero exposure time is always equal to 1 due to normalizing parasitemia to itself). Insets indicate the *in vitro* decay of DHA concentration. Empty circles display the raw viability data, and the curves pass through the arithmetic means of the paired data points. (Data are sourced from reference 7.)

To study stage-specific drug effects, Klonis et al. tested four different parasite ages (by using different age-synchronized groups): 2 h postinfection (h p.i.; early ring stage), 7.5 h p.i. (mid-ring stage), 24 h p.i. (early trophozoite stage), and 34 h p.i. (late trophozoite stage). Two examples of viability data are given in Fig. 1 and show the viability for different durations of drug exposure (1 h, 2 h, 4 h, and 6 h) with an initial DHA concentration of approximately 39 nM or 300 nM. Note that DHA concentration also decays *in vitro* with a half-life of approximately 8 h (which is much longer than that which occurs *in vivo*; see Discussion). Experiments were performed in technical replicates for each combination of initial DHA concentration and drug exposure duration.

We take as our fundamental conceptualization of antimalarial action that the drug kills or otherwise prevents parasites within infected red blood cells (iRBC) from being able to produce viable merozoites (which would go on to invade and infect other RBC at the end of the first life cycle). We model the number of iRBC of age *a* (i.e., RBC that have been infected with parasites for *a* hours) surviving drug exposure [*N*(*a*,*t*)] by the first-order partial differential equation

(3) $$\frac{\partial N(a,t)}{\partial t}+\frac{\partial N(a,t)}{\partial a}=-kN(a,t)$$

where *k* is a drug-induced parasite killing rate and may depend on other factors, such as drug concentration, parasite life stage, or even drug exposure duration (which will be explicitly indicated once we formally introduce those dependencies later). We have the boundary condition *N*(0,*t*) = *rN*(48,*t*), where *r* is the parasite multiplication factor, indicating the average number of newly infected RBC generated from merozoites released from a single iRBC at the end of the preceding life cycle.

The *in vitro* experiments use tightly age-synchronized parasites, allowing for further simplification to an ordinary differential equation system, which is sufficient for determining the time dependency in the maximum killing rate (*k*_max_) and the half-maximal killing concentration (*K_c_*). We track only the number of newly infected RBC generated from parasites first exposed to drug at age *ā* [denoted by *N̄*(*t*)]:

(4) $$\frac{d\overset{¯}{N}(t)}{dt}=-k\overset{¯}{N}(t)$$

As mentioned in the Introduction, the parasite killing rate, *k*, is empirically modeled by a Hill function of drug concentration, *C*(*t*),

(5) $$k(C(t))=\frac{k_{\text{max}}C{\left(t\right)}^{\gamma }}{{K_{c}}^{\gamma }+C{\left(t\right)}^{\gamma }}$$

where *k*_max_ is the maximum killing rate, *K_c_* indicates the drug concentration at which half-maximal killing (*k*_max_/2) is achieved and γ is the Hill coefficient. To capture the time-dependent features of the *in vitro* data, we generalized the model by allowing *k*_max_ and *K_c_* to be dependent on the duration of drug exposure. We considered the time variation to be a function of an auxiliary modulatory variable *S*(*t*), which we refer to as a general cell stress. During drug exposure, parasites develop a stress response, the extent of which determines the killing effect (and thus the concentration-killing rate function; equation 5). The stress, *S*(*t*), is normalized to vary between 0 and 1 (inclusive). We consider *S* to increase in the presence of drug above some (very small) threshold level, C*, but decrease once drug concentration, *C*, is below C*. For the increase phase, we apply a simple first-order differential equation:

(6) $$\frac{dS}{dt}=\lambda (1-S)$$

where λ is a rate constant which sets the time scale for stress development. In the absence of additional experimental data, *S*(*t*) is assumed to immediately reset to zero once drug concentration falls below C*. While this is sufficient to capture all available *in vitro* data, we anticipate that further experimental research will allow us to more closely tie empirical determinations of the mechanisms of stress and its accumulation to our modulatory variable, *S* (with consequential changes to equation 6).

We then assume that *k*_max_ and *K_c_* depend on the development of the stress response, i.e., they are functions of *S*. This construction allows for *k*_max_ and *K_c_* to vary with different drug exposure times, mediated through the stress response. *k*_max_ is evidently positively correlated with *S* and *K_c_* is negatively correlated, indicating that as stress accumulates, the ability of the drug (at a given concentration) to kill parasites increases. In the absence of detailed experimental data, we assume these relationships are linear:

(7) $$k_{\text{max}}=\alpha S$$

and

(8) $$K_{c}=\beta_{1}(1-S)+\beta_{2}$$

where α, β_1_, and β_2_ are parameters to be determined. α can be viewed as the maximum achievable killing rate (i.e., *k* = α), achieved only after long exposure to a high drug concentration. β_1_ affects how sensitive the killing rate, *k*, is to a change in the stress variable, *S*, and β_2_ represents the half-maximal activation concentration once stress is at a maximum (i.e., when *S* = 1). Note that to avoid issues of parameter identifiability as λ→∞, we do not allow for an additional constant term in the expression for *k*_max_. Similarly, and as explored further in Discussion, without direct experimental evidence for a particular underlying mechanism for stress, we have assumed that a single stress response is able to influence both *k*_max_ and *K_c_*.

Under this simple formulation of the model for stress accumulation and the linear relationship between stress and killing, we can solve equation 6 to obtain

(9) $$S(t)=1-e^{-\lambda t}$$

and thus

(10) $$k_{\text{max}}=\alpha (1-e^{-\lambda t})$$

and

(11) $$K_{c}=\beta_{1}e^{-\lambda t}+\beta_{2}$$

Of note, when λ→∞ (i.e., the modulatory variable reaches its steady state instantaneously), *k*_max_ = α and *K_c_* = β_2_ such that our model reduces to a traditional PK-PD model with a fixed relationship between drug concentration and killing rate.

**FIG 2:**
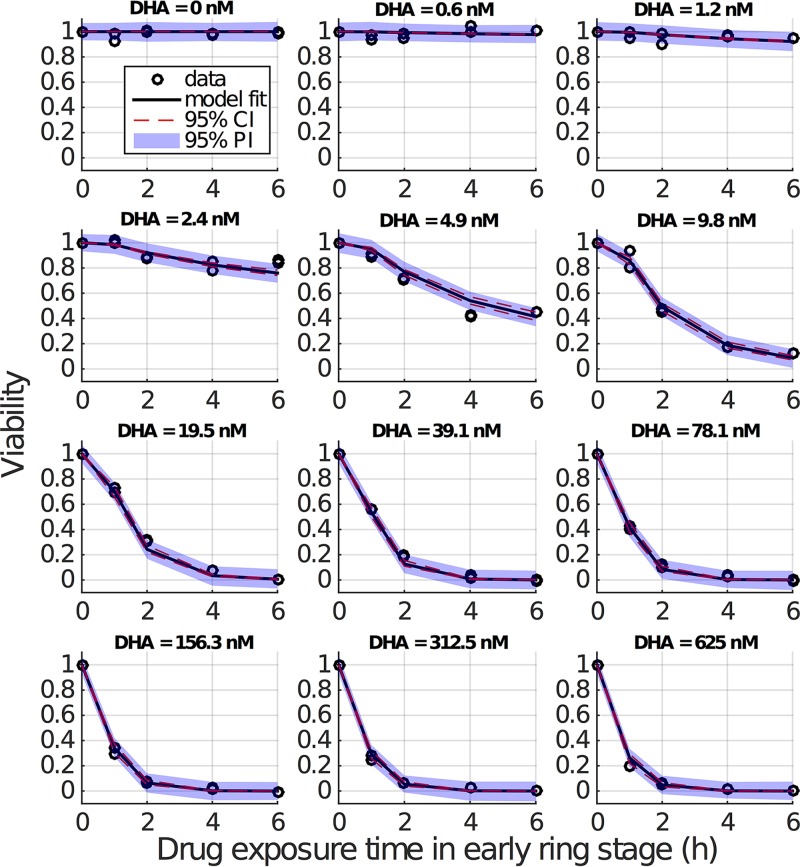
Results of fitting the model to viability data (early ring stage). The initially applied DHA concentration is indicated for each panel. Empty circles (appearing in duplicate) are the repeated measures of viability by (initial) drug concentration and exposure duration. For one DHA concentration and one drug exposure time in each panel, duplicate viability measurements are provided, which means in total 10 data points are measured in each panel (note that viability for zero exposure time is always equal to 1 due to normalizing parasitemia to itself). Black curves show the predicted mean viability measurements from the model with fixed γ parameter. Red dashed lines are the 95% confidence intervals (CI) for the predicted mean viability measurements (derived using simulation-estimation of 500 concentration-effect profiles and parametric bootstrap CIs), and blue-shaded regions are 95% prediction intervals (PI; derived 2.5th and 97.5th percentiles of 500 simulated concentration-effect profiles) for a new viability measurement if it were generated under the same experimental conditions (i.e., drug concentration and pulse duration).

For finite λ, *k*_max_ and *K_c_* become functions of *S* and so duration of exposure. In particular, for low λ, our model displays a slow development of the stress response and thus is capable of capturing a delayed reduction in viability, indicating its suitability for the *in vitro* data shown in Fig. 2 and detailed in references 7 and 9. For simplicity, in this paper we refer to the traditional killing rate model with constant (although perhaps stage-dependent) *k*_max_ and *K_c_* as the stationary model and refer to our generalized model as a dynamic stress model.

The dynamic stress model contains five parameters (λ, γ, α, β_1_, and β_2_) to be determined by fitting to available data. These five parameters are assumed to be stage specific, and thus the model is fitted separately to the viability data for each parasite stage. Note that by incorporating the phenomenological model of stress through the modulatory variable *S*, we aim to develop a better understanding of how the killing rate evolves in the presence of drug. While we are as yet unable to explore the underlying mechanisms governing the development of both stress and drug action (e.g., the changes at the cellular or even molecular levels), we discuss possible biological interpretations further in Discussion.

For modeling the *in vitro* experiments of tightly age-synchronized parasites, we use equation 4. The solution to equation 4, subject to an initial condition *N̄*(0) = *N̄*_0_, is

(12) $$\overset{¯}{N}(t)=\overset{¯}{N_{0}}e^{-\int_{0}^{t}k(C(\tau ),S(\tau ))d\tau }$$

where we have explicitly presented the killing rate, *k*, as a function of DHA concentration, *C*, and stress, *S*, both of which are functions of time (following initiation of drug exposure). DHA concentration, *C*(*t*), as a function of time due to *in vitro* decay, is given by

(13) $$C(t)=C_{0}e^{-\frac{\ln(2)}{t_{1/2}}t}$$

where *C*_0_ is the initial dug concentration and the *in vitro* half-life of DHA (*t*_1/2_) was measured to be about 8 h (21).

For a drug pulse with a duration of *t_d_* hours in a given stage of the parasite life cycle, the total number of iRBC, *N_d_*, at the time of data collection (during the trophozoite stage in the next life cycle) is given by

(14) $$N_{d}=r\overset{¯}{N_{0}}e^{-\int_{0}^{t_{d}}kd\tau }+\overset{¯}{N_{0}}\left(1-e^{-\int_{0}^{t_{d}}kd\tau }\right)$$

where *k*'s dependence on *C*(τ) and *S*(τ) is now implicit. The first term represents the number of iRBC with live parasites (having expanded by factor *r*, the parasite multiplication factor), while the second term represents the number of nonviable parasites. For the control case (no drug), the number of parasites, *N_c_*, is given by equation 14 with *k* = 0 (*N_c_* = *r**N*_0_). For the background case (all parasites killed due to supermaximal exposure), the number of parasites, *N_b_*, is given by equation 14 with *k*→∞ (*N_b_* = *N*_0_). Substituting these two expressions back into equation 14, we have

(15) $$N_{d}=N_{c}e^{-\int_{0}^{t_{d}}kd\tau }+N_{b}\left(1-e^{-\int_{0}^{t_{d}}kd\tau }\right)$$

which can be rearranged to give

(16) $$e^{-\int_{0}^{t_{d}}kd\tau }=\frac{N_{d}-N_{b}}{N_{c}-N_{b}}$$

The right-hand side is precisely the parasite viability (*V*) as defined in the *in vitro* experiments (7, 9). Therefore, we have

(17) $$V(C_{0},t_{d})=e^{-\int_{0}^{t_{d}}k(C(\tau ),S(\tau ))d\tau }$$

where the dependence of *V* on the initial DHA concentration, *C*_0_, is established through equation 13. Furthermore, we identify $\phi =\int_{0}^{t_{d}}k(C(\tau ),S(\tau ))d\tau$ as the cumulative stress effect. We have identified the parasite viability as a function of the cumulative stress effect arising from a dynamic mechanistic model, allowing us to fit our model to available viability data, and then use the estimated parameters to perform detailed PK-PD simulations in an *in vivo* context.

### Fitting the model to viability data.

Figure 2 shows the fitting result for the early ring stage. Results for the other stages are provided in Fig. S1 to S3 in the supplemental material. Parameter estimates and confidence intervals (CI) are given in Table 1. The model captures the data very well, in particular the dependence of viability on drug exposure time, which is the key advance we require. Note, as discussed in the introduction, the standard model (in which there is no stress accumulation) is incapable of explaining the experimental observations for short exposure times. The standard model always predicts a faster decrease in viability for a higher drug concentration, contradicting the data (Fig. 1). This is further confirmed by a statistical comparison of the dynamic stress model and the standard model both in terms of fit visualization (Fig. S4 to S7) and the Akaike information criterion (AIC) (Tables S2 and S3). In particular, the improvement of goodness of fit is most evident for the mid-ring stage (Fig. S5). This finding comes as no surprise; the estimates for λ for the mid-ring stage are small relative to the λ→∞ value, which recovers the standard (no stress accumulation) model. This analysis demonstrates that the introduction of a time-dependent stress response, modulating both the maximal killing rate and the half-maximal drug concentration, provides a substantially improved explanation for the *in vitro* experimental data.

**TABLE 1:** Results of fitting the model to viability data^*a*^

Parameter (unit)	Estimate	SE	95% CI	
			Model based	Parametric bootstrap
Early ring stage				
λ (h^−1^)	6.2504 (0.11)	0.5745	5.1243, 7.3765	1.8587, 9.5201
α (h^−1^)	1.6915	0.1378	1.4215, 1.9616	1.2944, 1.8475
β_1_ (nM)	990.84	373.49	258.81, 1,722.9	−20,377, 1876.5
β_2_ (nM)	12.519	1.0631	10.435, 14.602	10.560, 13.553
Mid-ring stage				
λ (h^−1^)	0.3729 (1.86)	0.1406	0.0974, 0.6485	0.2515, 0.5035
α (h^−1^)	1.1224	0.2455	0.6412, 1.6036	0.6934, 1.3371
β_1_ (nM)	224.39	112.12	4.6466, 444.14	117.37, 301.69
β_2_ (nM)	9.97 × 10^−4^	1.26 × 10^−4^	(7.5, 12.4) × 10^−4^	(9.8, 10.1) × 10^−4^
Early trophozoite stage				
λ (h^−1^)	1.2290 (0.56)	0.2249	0.7882, 1.6698	0.6331, 1.8059
α (h^−1^)	5.7434	0.7460	4.2813, 7.2054	1.8799, 7.2729
β_1_ (nM)	317.64	86.143	148.80, 486.48	−9.4144, 462.98
β_2_ (nM)	39.570	4.6038	30.546, 48.593	29.315, 46.842
Late trophozoite stage				
λ (h^−1^)	2.0906 (0.33)	0.2909	1.5203, 2.6608	1.4406, 2.6302
α (h^−1^)	2.8626	0.1591	2.5508, 3.1744	2.2851, 3.2810
β_1_ (nM)	740.02	178.77	389.64, 1,090.41	160.00, 1,071.6
β_2_ (nM)	41.405	3.6606	34.230, 48.580	35.459, 45.629

a Viability data are for the 3D7 strain (7). γ is fixed to be 1.7892 based on estimates in Table S1 in the supplemental material. The model-based 95% CI and parametric bootstrap 95% CI are introduced in Materials and Methods. The rate of stress development, λ, can also be understood as the rate at which the “unstress” state is lost, and the half-life (hours) of the unstressed state can be calculated simply by ln(2)/λ and is shown in parentheses following the estimates of λ.

We also compare the model fits on a logarithmic scale (Fig. S8 to S11). The dynamic stress model outperforms the standard model overall, particularly so for low concentrations and short exposure times, which are the primary point of interest. However, for some cases of high DHA concentration and long exposure times (e.g., for 4-h and 6-h exposures at drug concentrations larger than 100 nM), we identify that the dynamic stress model subtly overestimates the killing effect for early ring and early and late trophozoite stages (Fig. S8, S10, and S11). Of course, the log scale emphasizes the subtle effect while obscuring the significantly improved fit for shorter exposure durations and lower drug concentrations (where the interesting and new dynamic effects are evident). Furthermore, the limit of detection in the *in vitro* experiment remains uncertain, and while we conservatively assumed a detection limit of 0.005 for data fitting, it may be as high as 0.05. Refitting with a higher detection limit (of 0.05) provides nearly indistinguishable best fits to the data with no meaningful change in parameter estimates (data not shown) but does provide even stronger statistical support for the dynamic stress model over the standard model given the relatively diminished contribution to the likelihood from low-viability data (more of which now falls below the detection threshold).

### Drug concentration-killing rate curves and stage dependency.

**FIG 3:**
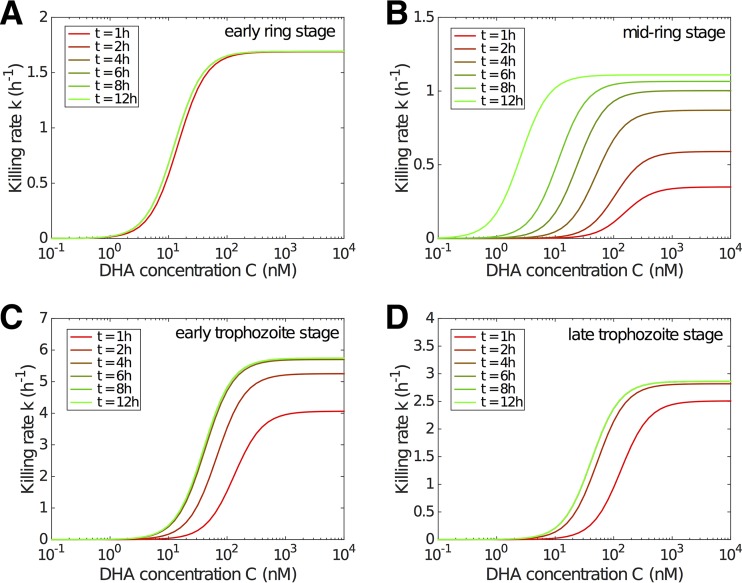
Model results showing the evolution of the drug concentration-killing rate curve with drug exposure duration for different stages. The time after drug exposure, *t*, is indicated. Note that the *y* axis scale differs for different stages.

The overall impact of parasite killing is determined primarily by the drug concentration-killing rate curve, which we now consider to be a function of exposure time, generalizing the usual modeling assumption that the killing rate is an instantaneous function of drug concentration. Figure 3 shows the modeled evolution of the concentration-killing rate curve for the four different life stages of the parasites used in Fig. 1. Except for the early ring stage, for which the curve reaches its steady state very quickly, the delayed process of approaching the steady-state killing rate curve for the other three stages is biologically significant, particularly for the mid-ring stage where a very strong delay is observed.

**FIG 4:**
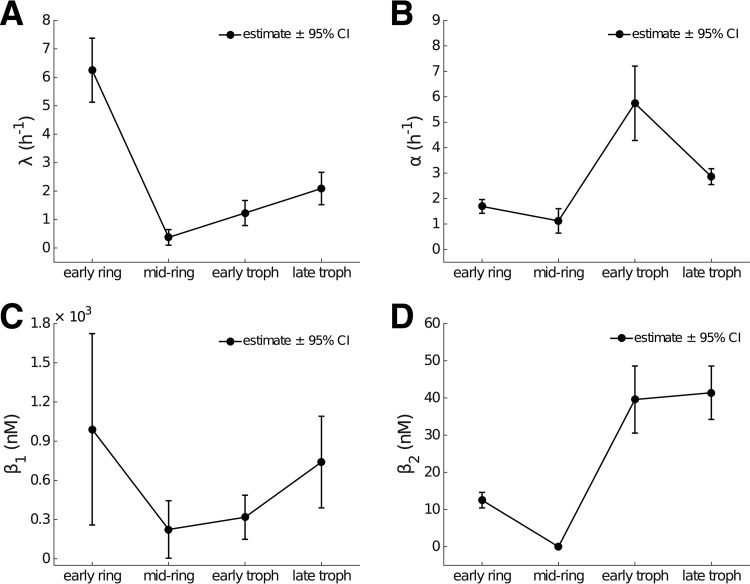
Dependence of model parameters on parasite life stage. The parameter estimates are provided in Table 1. Error bars show the model-based 95% CI for each parameter. (A) The rate of stress development (λ) is large for early rings, indicating minimal time dependence in killing for this life stage. In contrast for mid-ring-stage parasites, the rate is very small, indicating a substantial accumulation effect. (B) The maximum killing rate is linear in α, indicating that the maximum killing rate is higher in trophozoites than rings. (C and D) Parameters (β_1_ and β_2_) model the relationship between stress (*S*) and the half-maximal drug concentration (equation 8).

Figure 4 shows the estimates and 95% confidence intervals for the four model parameters λ, α, β_1_, and β_2_ by stage. The delay in the evolution of the drug concentration-killing rate curve is determined primarily by the parameter λ (Fig. 4A and Table 1), which reflects the accumulation rate for stress (equation 6). For the early ring stage, λ = 6.25 h^−1^ and thus *S*(1 h) > 0.99, indicating that early rings rapidly succumb to drug exposure. In contrast, the rate of accumulation of stress for mid-ring-stage parasites is much lower (λ = 0.37 h^−1^), and it would take over 12 h of continued exposure to drug for *S* to exceed 0.99. Early and late trophozoite stages display similar characteristics in terms of the rate of accumulation of stress (Fig. 3). The temporal effect can also be interpreted by introducing the half-life (Table 1) of the unstressed state, given by ln(2)/λ. Accordingly, λ can be seen as the rate at which the unstressed state is lost. We see that the stress in mid-ring-stage parasites takes approximately 2 h to reach 50%, while stress accumulates for other stages more rapidly, taking less than half an hour to reach the same level.

The maximum killing rate, α (Fig. 4B), shows a significant reduction for (early and mid-) ring-stage parasites than for (early and late) trophozoites, suggesting that young parasites are more resistant to DHA than mature parasites once (or even when) the killing effect has reached a steady state. The parameters related to the concentration required to achieve the half-maximal killing rate, β_1_ and β_2_, also exhibit stage specificity (Fig. 4C and D). In particular, the stationary half-maximal killing concentration (Fig. 4D, β_2_) shows that ring-stage parasites exhibit a higher sensitivity to drug at steady state than trophozoites. However, it must be remembered that, particularly for mid-ring-stage parasites, the progress toward that steady state (governed by λ) is slow, and the net effect of the dynamics of drug-induced killing is best understood through Fig. 3.

### Incorporating the delayed drug effect into PK-PD modeling.

Having established the applicability of our model to the *in vitro* data, we now consider the potential implications for the *in vivo* application of artemisinin-based medication. We do so by incorporating the time dependency on the killing rate into the general PK-PD framework (equation 3), where we allow for realistic drug PK and a general age structure for the parasites.

**FIG 5:**
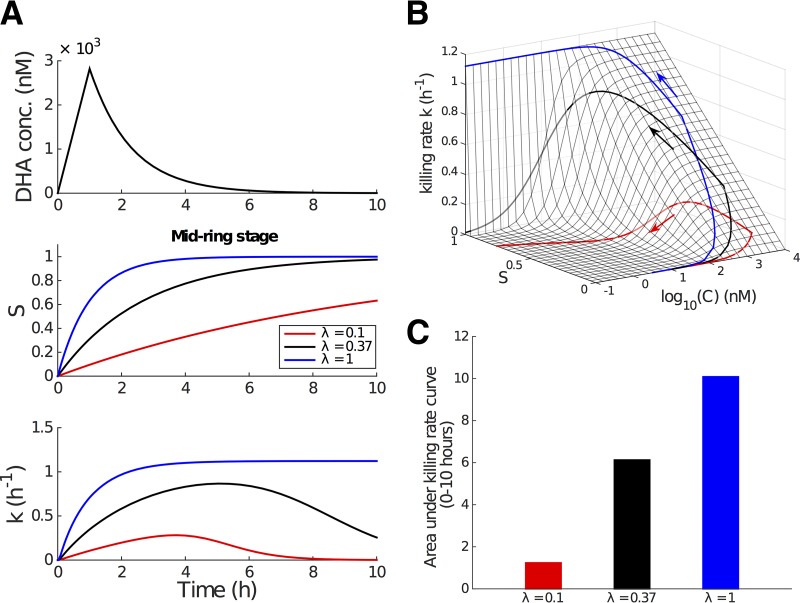
Incorporation of the time-dependent killing rate into the PK-PD model. We study the mid-ring stage for illustrative purposes. Parameter values are taken from Table 1. (A) Simulated *in vivo* DHA concentration profile (upper; the *in vivo* half-life is approximately 0.9 h [2, 21]), the kinetics for the modulatory stress variable, *S* (middle; black curve, λ = 0.37), and the transient killing rate *S* (lower; black curve, λ = 0.37) induced by the drug pulse. The middle and lower panels also show how *S* and *k* evolve if λ is higher (blue) or lower (red). (B) Killing rate surface as a function of DHA concentration, *C*, and the stress, *S*, and the projection of the trajectory of the effective killing rate (i.e., a projection of the curves in panel A [lower]) onto the surface. (C) Area under the killing rate curve, an indication of the total amount of killing achievable over the course of the drug pulse.

Because the stress accumulation effect is most pronounced for the mid-ring stage of 3D7 parasites (the subject of our study), we begin by considering mid-ring-stage parasites treated with a single dose of artesunate (2 mg/kg of body weight). The plasma DHA concentration, *C*(*t*), displays biphasic behavior (6):

(18) $$\frac{dC}{dt}=\left\{ \begin{array}{l} \frac{C_{\text{max}}}{t_{m}},0\leq t<t_{m}\\ -\frac{\ln(2)}{t_{1/2}}C,t\geq t_{m} \end{array}\right.$$

where *C*_max_ is the maximum achievable concentration and *t_m_* indicates the time at which that maximum concentration is achieved. Note that the half-life, *t*_1/2_, refers to the *in vivo* half-life of DHA, which is much smaller than that measured *in vitro* due to altered physiological conditions (Fig. 2 and Table S2 in reference 21), i.e., *C*_max_ of 2,820 nM, *t_m_* of 1 h, and *t*_1/2_ of 0.9 h per references 2 and 21. The simulated PK data are shown in Fig. 5A, upper.

The middle and lower panels of Fig. 5A shows the time series for stress, *S*, and killing rate, *k*, as a result of the changing DHA concentration. The black curves are generated using λ = 0.37 h^−1^, the best-fit estimate from fitting the model to the *in vitro* data for the mid-ring stage (Table 1). Decreasing λ will delay the increase of *S* and in turn lead to a slower and shortened killing rate profile (Fig. 5A, red curves), while increasing λ will do the opposite (Fig. 5A, blue curves). Thus, we consider a lowering of λ as a potential manifestation of (stage-specific) artemisinin resistance. Conversely, λ = 1 implies that parasites accumulate stress rapidly and are rendered nonviable at the stationary rate [given *C*(*t*)] soon after initiation of the drug pulse.

Under the model, the killing rate, *k*, is now a function of two variables (as clearly shown by equation 17), the drug concentration, *C*(*t*), and the modulatory stress, *S*(*t*). We can represent this graphically by displaying the killing rate as a trajectory on a surface in (*C*,*S*) space (Fig. 5B). In this representation, we can clearly see the effect of λ on the evolution of the killing rate: the trajectory corresponding to a smaller λ has less time (controlled by *S* in the model) to climb up the killing rate surface, even when the achievable DHA concentration remains the same.

We can also consider the net cumulative effect of the drug pulse. As described earlier, $\phi =\int_{0}^{t_{d}}k(C(\tau ),S(\tau ))d\tau$ represents the cumulative stress effect, which can be used to indicate drug efficacy. In the *in vivo* simulation, ϕ is simply the area under the effective killing rate curve (i.e., the area under the curve in the lower panel of Fig. 5A). For mid-ring-stage parasites with λ = 0.37, the cumulative stress effect (Fig. 5C, black bar) corresponds to a reduction in viability of approximately 99.75% over the drug pulse. Further numerical exploration indicates that a roughly 3-fold increase or decrease in λ leads to a significant difference in the cumulative stress effect (Fig. 5C, blue and red bars) and, in turn, a difference of a few orders of magnitude in viability.

In summary, the results presented in Fig. 5 indicate that the temporal drug effect significantly affects the *in vivo* parasite killing and thus should be considered in model-based prediction of clinical treatment. Furthermore, the visualization of the killing rate trajectory on the (*C*,*S*) plane surface suggests a clear evolutionary strategy for the parasite to escape drug pressure, particularly given the short elimination half-life of artemisinin and its derivatives. An ability to outlast the short drug pulse provides an effective means of escape quite distinct from any changes in susceptibility, as are typically considered by a change in the maximal killing rate or drug concentration required to achieve half-maximal killing.

**FIG 6:**
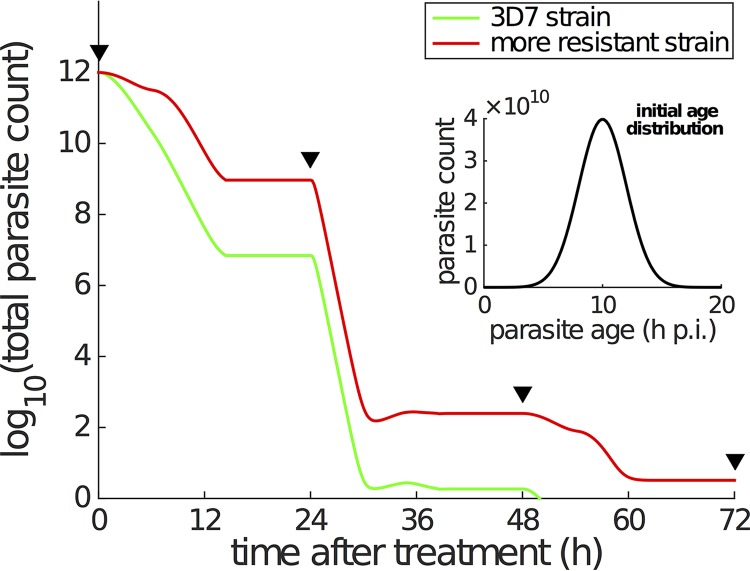
Simulation of parasite killing under a standard treatment of 2 mg/kg artesunate every 24 h. The inset shows the age distribution of a total of 10^12^ parasites (per patient) at the start of treatment [∼*N*(10, 2^2^)]. The parasite multiplication factor is assumed to be 10 (6, 13), which means that 10 new parasites are produced once a parasite reaches 48 h p.i. (i.e., *r* = 10 in the model). The PK profile is a series of repeated DHA concentration profiles every 24 h (i.e., repeated simulations of DHA concentration profile shown in Fig. 5A, upper). The black triangles indicate when the doses are given. The green curve corresponding to the laboratory 3D7 strain is generated using the parameters in Table 1, while the red curve is generated using the same set of parameters except for reducing λ for mid-ring stage to be 0.1 h^−1^ to simulate a more resistant strain. With limited information, we simply divide the 48-h life cycle into early ring stage (0 to 6 h p.i.), mid-ring stage (6 to 26 h p.i.), early trophozoite (26 to 34 h p.i.), and late trophozoite (34 to 48 h p.i.) in the simulation. The modulatory variable *S* is assumed to follow equation 6 only when DHA concentration, *C*, is ≥0.1 nM (i.e., C* = 0.1 nM), and *S* is immediately reset to zero when the DHA concentration drops below 0.1 nM.

To fully explore the consequences of accumulation effects on the pharmacodynamics of antimalarial treatment, we simulated the time course of total viable parasite count under a standard AS7 dosing regimen (i.e., a dose of 2 mg/kg artesunate every 24 h for 7 days) for both the 3D7 strain and a hypothetical strain which exhibits a slower rate of stress development during the mid-ring stage. Some key simulation details are provided in the legend to Fig. 6. We initiated the simulation with 10^12^ parasites per patient with a normally distributed age distribution, with a mean of 10 h p.i. and standard deviation of 2 h p.i. (Fig. 6, inset). For the laboratory 3D7 strain, the model predicts that effective parasite clearance is achieved immediately following the third dose of artesunate (at 48 h in the model) (Fig. 6, green curve). In contrast, for the hypothetical strain which exhibits a slower development of the stress response during the mid-ring stage (i.e., λ is reduced for this, but no other, stage), we observe a clear and substantial delay in parasite clearance. In detail, the red curve in Fig. 6 shows the parasitemia curve for a resistant strain that has a λ value of 0.1 h^−1^ for the mid-ring stage (with all other parameters [across all stages] unchanged). This simulation has an *a priori* rationale given previous studies that indicate that field isolates from Pailin (western Cambodia) display a reduced sensitivity to artemisinin-based therapies during the ring stage of infection (6, 8, 9). We note that while this simple simulation does not incorporate the process of splenic clearance or the immune response (which are also important for *in vivo* parasite clearance; see Discussion), its behavior is consistent with clinical observations of a 1.5 to 2 times longer time to clearance for resistant strains compared to sensitive strains (6).

In addition, we also compare the *in vivo* simulation result generated by using the standard killing rate model to the results shown in Fig. 6 (i.e., the results generated by using the dynamic stress model) in Fig. S12. The two models produce qualitatively similar results. Parasites are killed in a stepwise manner associated with the time of drug administration. However, the stress model predicts a much faster parasite clearance than the standard model. This is due to the different ways in which the dynamic stress and traditional models explain the *in vitro* data. Without an accumulation effect, the standard model does its best to explain the *in vitro* data by having a lower maximal killing rate (of course, this leads to poor fits compared to the dynamic stress model, so little confidence should be placed in the extrapolated *in vivo* parasite-time curve). In turn, the lower estimates for *k*_max_ compared to α (and higher estimates for *K_c_* compared to β_2_) lead to slower parasite killing in the *in vivo* context. We note that, like for all PK-PD studies that attempt to translate from the *in vitro* to *in vivo* context, care must be taken as the *in vivo* environment may result in quantitative changes to the values of key model parameters (13). Accordingly, the key finding from our *in vivo* simulation is a qualitative, not quantitative, one. We have demonstrated how a change in the rate of accumulation of stress may influence the clearance time and how ignoring stress may provide vastly different predictions for the clearance time. Conducting such a quantitative translation exercise to the *in vivo* context using the techniques of Zaloumis et al. (13) will be the subject of future work.

## DISCUSSION

Artemisinin resistance has arisen as the major impediment to the continued success of malaria control programs. With new drugs likely to be some time away from licensure and widespread use, how we maintain the effectiveness of artemisinin-based therapies is an important and urgent problem to resolve. In this paper, we have introduced a novel mathematical model that allows for the detailed investigation of the time-dependent response of P. falciparum parasites to the artemisinins. We have established the significant influence of the parasite stress response on killing and incorporated this into a simulation of *in vivo* parasite clearance. This is the first study to our knowledge to incorporate the novel time-dependent drug effect into a fully mechanistic PK-PD model, and it constitutes an essential step toward development of a comprehensive framework that can be used to optimize existing dosing regimens.

Validated against detailed *in vitro* experimental data (7, 9), the key feature introduced in our model is the concept of dynamic (accumulating) stress (*S*) and the parameter governing the time scale of that process (λ). The time evolution of stress determines the development of the killing rate and therefore the probability of parasite survival (as assessed *in vitro* by viability). This conceptualization of stress, applied to the artemisinin derivatives, has been shown to not only capture the *in vitro* viability data published in reference 7 but also identify the relative contributions of drug concentration and stress response in determining the effective killing effect.

Specifically, the estimate for λ, which determines the strength of the delay, shows that mid-ring-stage 3D7 parasites exhibit a substantially more delayed response to DHA exposure than do parasites at other stages. This is consistent with the original analyses presented in Klonis et al. (7), where a semimechanistic (but not dynamic) cumulative effective dose (CED) model was used for interpretation of stage-specific drug effects. Klonis et al. also reported an unexpected finding that early-ring-stage parasites exhibited a hypersensitivity to DHA compared to mid-ring-stage parasites. Our model results (Fig. 4) show that a rapid induction of maximal killing (i.e., a rapid increase in *S*), rather than a large increase in the magnitude of the maximum killing rate itself, is the primary explanation for this hypersensitivity.

We also examined, through simulation of the full PK-PD model, how a cumulative stress response affects *in vivo* parasite dynamics. We considered the standard AS7 artesunate treatment regimen (a dose of artesunate every 24 h for 7 days). Parasites that are more able to withstand exposure (i.e., that have a lower λ) during the mid-ring stage remain in circulation for a significantly greater period of time, with PD profiles that reflect those from patients infected with resistant strains. These results agree with those we have previously presented using the CED model (see Fig. 7 in reference 9), but we emphasize that the results presented here (Fig. 6) arise from a fully mechanistic PK-PD model. This is an important distinction as, by construction, our PK-PD model accounts for the ageing and natural replication dynamics of the parasite population, the time-varying nature of the drug concentration, and the interaction (killing) between parasite and drug in a self-consistent and biologically realistic way. This provides our model with enhanced predictive power compared to the CED model, which, while empirically useful, was not well suited to *in vivo* simulation. We emphasize that our *in vivo* simulations provide predictions of the number of viable parasites. However, *in vivo* assays cannot distinguish between viable and nonviable parasites or detect sequestered parasites. As such, further advances in experimental assays are required to fully test the predictions from these simulations.

We have referred to the modulatory variable, *S*, throughout the paper as a stress. We have done so to provide guidance as to possible biological interpretations of *S*, but for now an incomplete understanding of the mechanism of action of the artemisinins limits the degree to which our phenomenologically based model can be correlated with specific biological stresses induced by exposure to artemisinins. Recent work (22, 23) confirms earlier studies (24) suggesting that artemisinins exert their activity by alkylating multiple targets within the parasite. Reports of growth retardation, quiescence, and dormancy following artemisinin exposure (7, 9, 25, 26) are reminiscent of the cytostatic stress response observed in other organisms (27, 28). Further developments in understanding the mechanistic underpinnings of artemisinin activity are required to further refine our model for stress. The details of any of these processes, were they to be confirmed to be associated with cumulative stress effects, would be able to be incorporated into our model in a straightforward manner through adjustment of the equations governing the time evolution of *k*_max_ and *K_c_*.

An immediate implication of our model concerns the possible mechanism by which the malaria parasite attains resistance to artemisinin. Drug resistance is typically characterized by an increase in the drug concentration required (*in vitro* or *in vivo*) to achieve maximal (or half-maximal) killing. However, our exploratory analysis suggests that increasing tolerance to stress (i.e., reducing λ) can also underpin drug escape. Indeed, the experimental results from reference 9, in which resistance can be overcome through application of proteasome inhibitors, such as carfilzomib, support this possibility. Furthermore, if such a mechanism were at play, then long-lived drugs acting on the same (or a similar) pathway and subject to the same resistance mutations would not result in a resistance phenotype when applied for extended periods (as *S* would still saturate and a high rate of killing would be achieved). This is precisely the behavior observed for OZ439 (half-life over 10 h) in recent experiments (21).

We also emphasize that the purpose of this study was not to determine the optimal structure (from a statistical perspective) for a model of stress accumulation, because the *in vitro* data do not provide any direct insight into the underlying mechanisms of stress. Rather, based on an analysis of the *in vitro* data, we established (i) the necessity for generalizing the standard killing rate model to capture the novel exposure time-dependent killing effect and (ii) the potential importance of the temporal effect in *in vivo* parasite clearance. To achieve this, it was sufficient to choose a simple mechanistic model that is able to explain the available viability data and that is statistically superior to the standard model. The novelty of our model lies not in its particular functional form but in its ability to capture the time dependency in exposure and killing.

The model we have introduced, while overcoming restrictions of the standard PK-PD approach and successfully capturing the complex dynamics observed in reference 7 for the 3D7 strain, has a number of limitations. Most importantly, equation 6 assumes a drug concentration-independent increase in *S*, while *C* is greater than C*, and then an instantaneous return to zero when the drug concentration drops below C*. However, this simple approach to modeling the stress, *S*, reflects the current limitations on our understanding of the mechanism of action of the artemisinins. With further data on how the drugs act, the dynamics of stress in the model can be adjusted to reflect the improved understanding. One important avenue to pursue is to examine how parasites that survive exposure to an initial drug pulse respond to a subsequent drug pulse. Does their stress (*S*) return to zero, or do they display some memory of previous exposure and thereby presumably succumb more quickly upon subsequent exposure? If such recovery exists, what is the typical time scale in relation to the life cycle? Such possibilities are (i) able to be probed experimentally using previously published techniques (7, 9) and (ii) able to be readily incorporated into more complex models of the form introduced in this paper. Moreover, it is possible that the killing parameters *k*_max_ and *K_c_* are modulated by distinct (or even multiple) stress responses. While such effects could be captured by a more flexible dynamic stress model by allowing parameters governing the time dependence for these two processes (i.e., λ) to be distinct, sufficiently detailed data are not yet available to justify exploration of such extensions. Another area for improvement in the approach taken here is in translating from the *in vitro* to *in vivo* situation. For example, our simple simulations assume there is no killing of parasites due to immune response mechanisms triggered within the host. While the immune response is unlikely to play a major role during the early stages of infection, as infection progresses its effects would be anticipated to become more significant. Therefore, given the fact that both the drug effect and immune response are dynamic in nature, it will be important to explore how differences in the timing of drug application and activation of various immune mechanisms affect parasite clearance and optimization of drug regimens. In the meantime, our results provide new insight into how P. falciparum responds to drug. Our model provides an enhanced predictive platform for evaluating the likely efficacy of alternative artemisinin-based drug regimens, directly contributing to the efforts to maintain effective control of malaria.

## MATERIALS AND METHODS

In this section, we introduce the statistical methods for model parameter estimation. Estimates of the model parameters (λ, γ, α, β_1_, and β_2_) for each parasite stage (early ring, mid-ring, early trophozoite, and late trophozoite) were obtained using nonlinear mixed-effect (NLME) modeling to fit equation 17 separately to the viability data for each stage. For each stage, the data with different drug concentrations were fitted simultaneously. (Note that the stage-dependent estimate of γ suggested a very limited variation [see Table S1 in the supplemental material] and thus was fixed later to be the mean of the four estimates in Table S1 to reduce uncertainty; main results in the paper were based on a fixed γ.) To account for the dependency between duplicate measurements, the residual error term was partitioned into between- and within-duplicate components that were assumed to be uncorrelated and normally distributed with means of zero and variances of $\sigma_{b}^{2}\;\text{and}\;\sigma_{w}^{2}$, respectively. The M3 method was used to account for viability data below the quantification limit of 0.005 (29).

Model-based 95% confidence intervals were calculated using asymptotic standard errors (square root of the inverse Fisher information) with an estimate of ±1.96× asymptotic standard errors. Ninety-five percent parametric bootstrap confidence intervals for the model parameters (Table 1 and Table S1) and predictions (Fig. 2 and Fig. S1 to S3) were calculated by (i) generating 500 parametric bootstrap data sets by simulating from the fitted NLME model; (ii) obtaining bootstrap estimates of the model parameters and predictions by refitting the NLME model to each parametric bootstrap data set; and (iii) calculating basic bootstrap confidence intervals for each parameter and prediction: 2 times the estimate minus the 97.5th percentile of bootstrap estimates and 2 times the estimate minus the 2.5th percentile of bootstrap estimates (30). Parametric prediction intervals for a new viability measurement (Fig. 2 and Fig. S1 to S3) were calculated at the observed pulse durations and DHA concentrations by (i) simulating 500 viability data sets from the fitted NLME model and (ii) calculating the 2.5th and 97.5th quantiles of the viability measurements simulated at each observed pulse duration and DHA concentration.

NONMEM 7.3.0 (ICON Development Solutions, Ellicott City, MD) and Perl-speaks-NONMEM 3.7.6 (31) were used to perform the NLME modeling of the viability data and obtain asymptotic standard errors and to perform the simulation-estimation procedure required to construct the 95% parametric bootstrap confidence intervals and the simulations necessary to calculate 95% parametric prediction intervals. MATLAB (version 2014b; The MathWorks, Natick, MA) was used to summarize and visualize the fitting results.

## Supplementary Material

Supplemental material
