# Supplementary material for: A Dynamic Stress Model Explains the Delayed Drug Effect in Artemisinin Treatment of Plasmodium falciparum
Source: Antimicrob Agents Chemother. 2017 Nov 22;61(12):e00618-17. doi: 10.1128/AAC.00618-17 (PMC5700357; doi:10.1128/AAC.00618-17)
Supplement: Supplemental material [file supp_61_12_e00618-17__index.html]

Supplemental material 

# A Dynamic Stress Model Explains the Delayed Drug Effect in Artemisinin Treatment of Plasmodium falciparum

## Supplemental material

- Supplemental file 1 -

  Supplemental Figures S1 to S12 and Tables S1 to S3

  PDF, 449K
