# Supplementary material for: A Dynamic Stress Model Explains the Delayed Drug Effect in Artemisinin Treatment of Plasmodium falciparum
Source: Antimicrob Agents Chemother. 2017 Nov 22;61(12):e00618-17. doi: 10.1128/AAC.00618-17 (PMC5700357; doi:10.1128/AAC.00618-17)
Supplement: Supplemental material [file AAC.00618-17_zac012176735s1.pdf]

Supplemental Material for "A dynamic stress model explains the delayed drug effect in artemisinin treatment of *Plasmodium falciparum*"

Pengxing Cao[1], Nectarios Klonis[2], Sophie Zaloumis[3], Con Dogovski[2], Stanley C. Xie[2], Sompob Saralamba[4], Lisa J. White[4], Freya J. I. Fowkes[3,5], Leann Tilley[2], Julie A. Simpson[3], and James M. McCaw*[1,3,6]

[1]School of Mathematics and Statistics, The University of Melbourne, Melbourne, Australia.
[2]Department of Biochemistry and Molecular Biology and Australian Research Council Centre of Excellence for Coherent X-Ray Science, Bio21 Molecular Science and Biotechnology Institute, University of Melbourne, Melbourne, Australia.
[3]Centre for Epidemiology and Biostatistics, Melbourne School of Population and Global Health, The University of Melbourne, Melbourne, Australia.
[4]Mahidol-Oxford Tropical Medicine Research Unit, Faculty of Tropical Medicine, Mahidol University, Rajthevee, Bangkok, Thailand.
[5]Burnet Institute, Melbourne, Australia.
[6]Modelling and Simulation, Infection and Immunity Theme, Murdoch Childrens Research Institute, The Royal Children's Hospital, Parkville, Victoria, Australia.

*Correspondence: jamesm@unimelb.edu.au

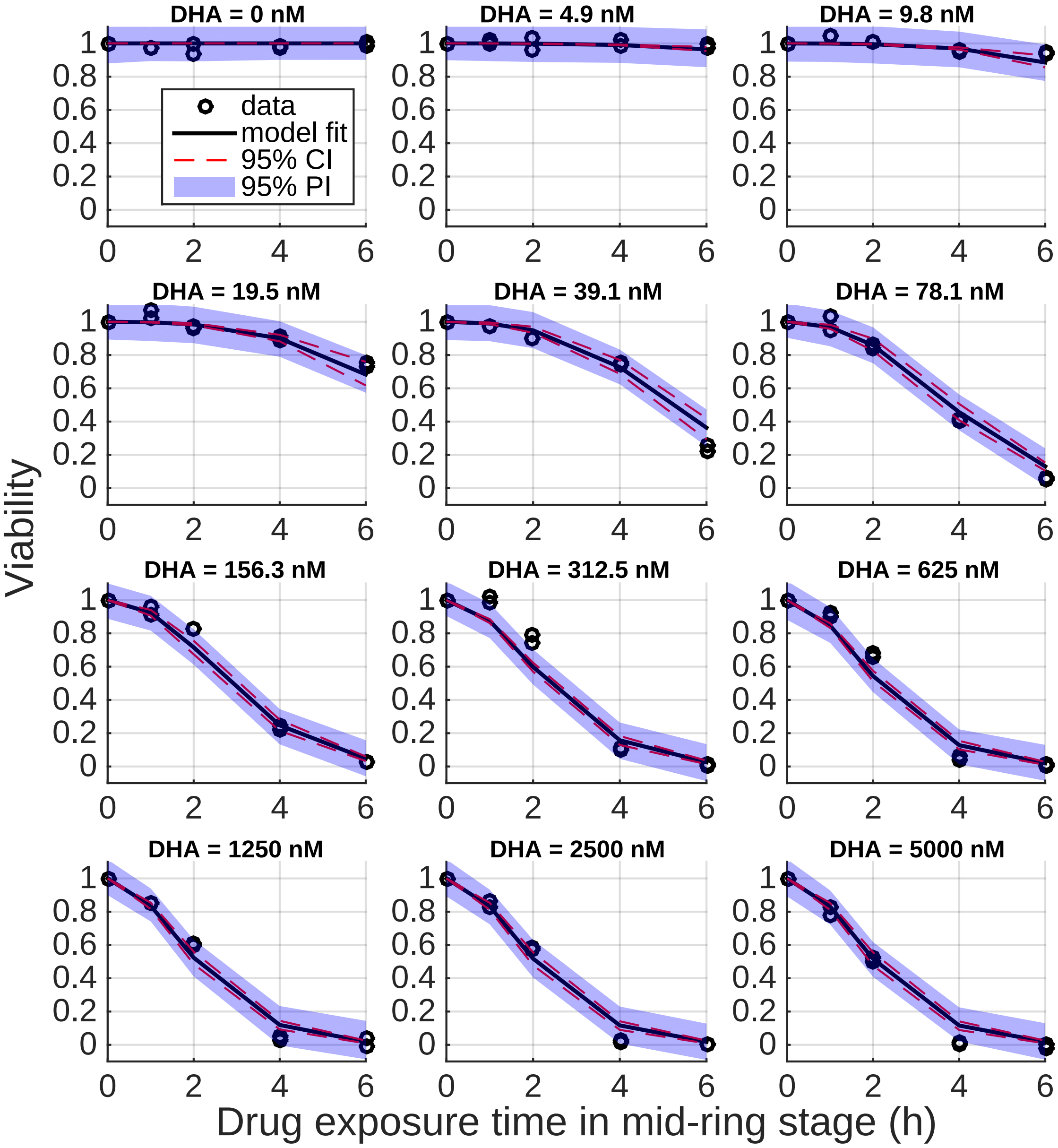

Figure S1: Results of fitting the model to viability data (mid-ring stage). The initially applied DHA concentration is indicated in the title of each panel. Empty circles (appearing in duplicate) are the repeated measures of viability by (initial) drug concentration and exposure duration. Black curves show the predicted mean viability measurements from the model with fixed γ parameter. Red dashed lines are the 95% confidence intervals (CI) for the predicted mean viability measurements (derived using simulation-estimation of 500 concentration-effect profiles and parametric bootstrap CIs) and blue shaded regions are 95% prediction intervals (PI; derived 2.5^{th} and 97.5^{th} percentiles of 500 simulated concentration-effect profiles) for a new viability measurement if it were generated under the same experimental conditions (i.e. drug concentration and pulse duration).

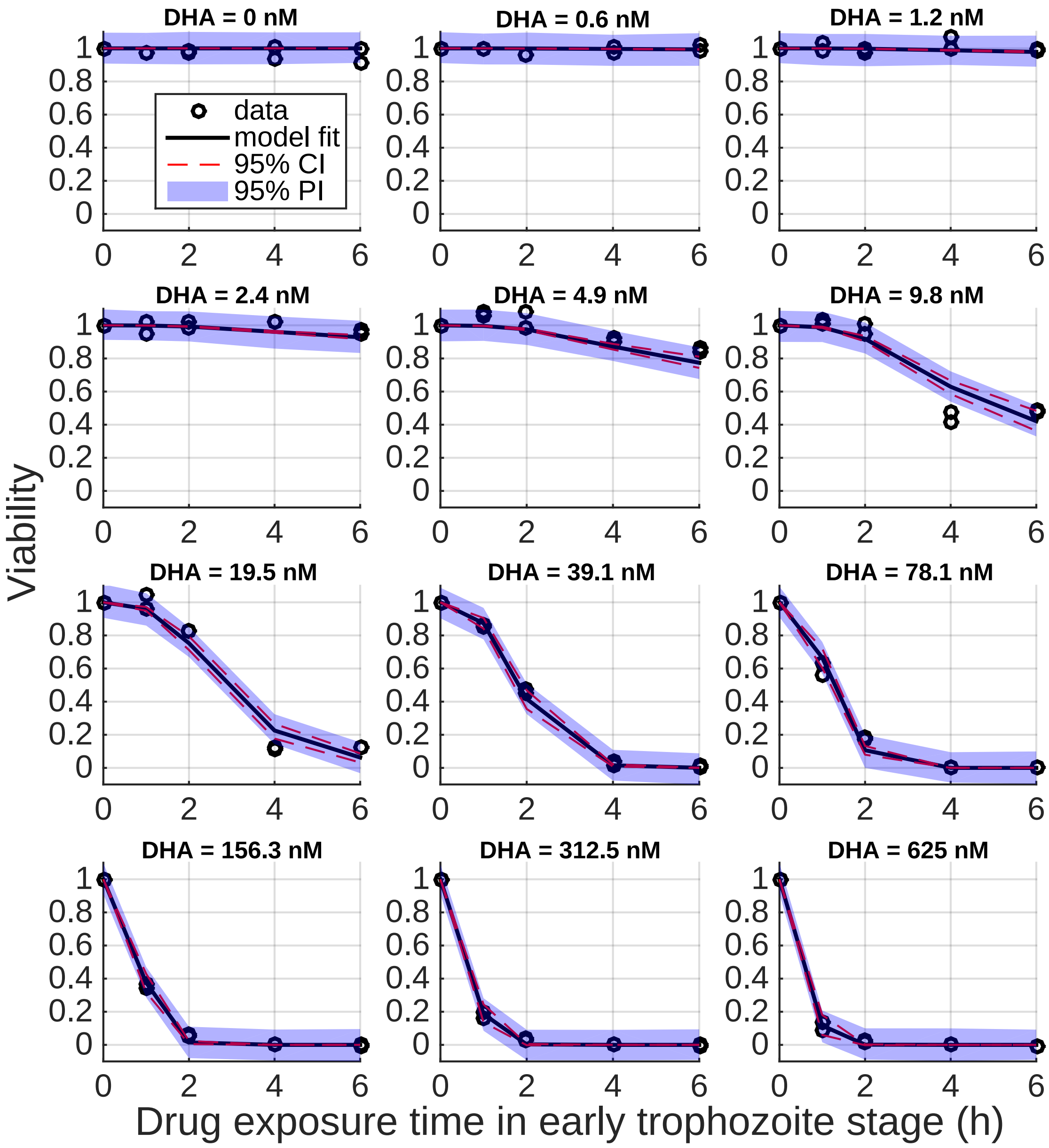

Figure S2: Results of fitting the model to viability data (early trophozoite stage). The initially applied DHA concentration is indicated in the title of each panel. Empty circles (appearing in duplicate) are the repeated measures of viability by (initial) drug concentration and exposure duration. Black curves show the predicted mean viability measurements from the model with fixed γ parameter. Red dashed lines are the 95% confidence intervals (CI) for the predicted mean viability measurements (derived using simulation-estimation of 500 concentration-effect profiles and parametric bootstrap CIs) and blue shaded regions are 95% prediction intervals (PI; derived 2.5^{th} and 97.5^{th} percentiles of 500 simulated concentration-effect profiles) for a new viability measurement if it were generated under the same experimental conditions (i.e. drug concentration and pulse duration).

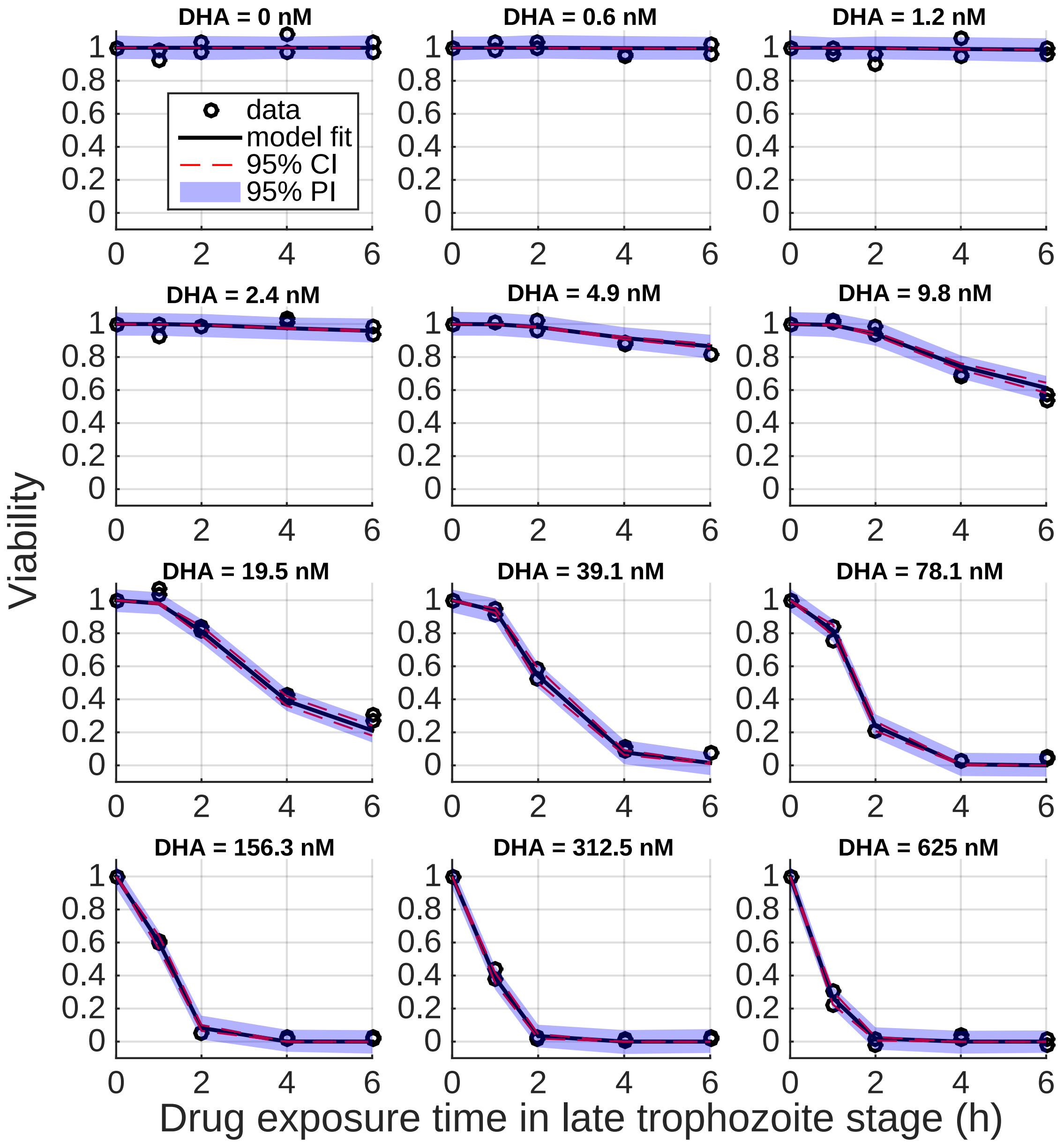

Figure S3: Results of fitting the model to viability data (late trophozoite stage). The initially applied DHA concentration is indicated in the title of each panel. Empty circles (appearing in duplicate) are the repeated measures of viability by (initial) drug concentration and exposure duration. Black curves show the predicted mean viability measurements from the model with fixed γ parameter. Red dashed lines are the 95% confidence intervals (CI) for the predicted mean viability measurements (derived using simulation-estimation of 500 concentration-effect profiles and parametric bootstrap CIs) and blue shaded regions are 95% prediction intervals (PI; derived 2.5th and 97.5th percentiles of 500 simulated concentration-effect profiles) for a new viability measurement if it were generated under the same experimental conditions (i.e. drug concentration and pulse duration).

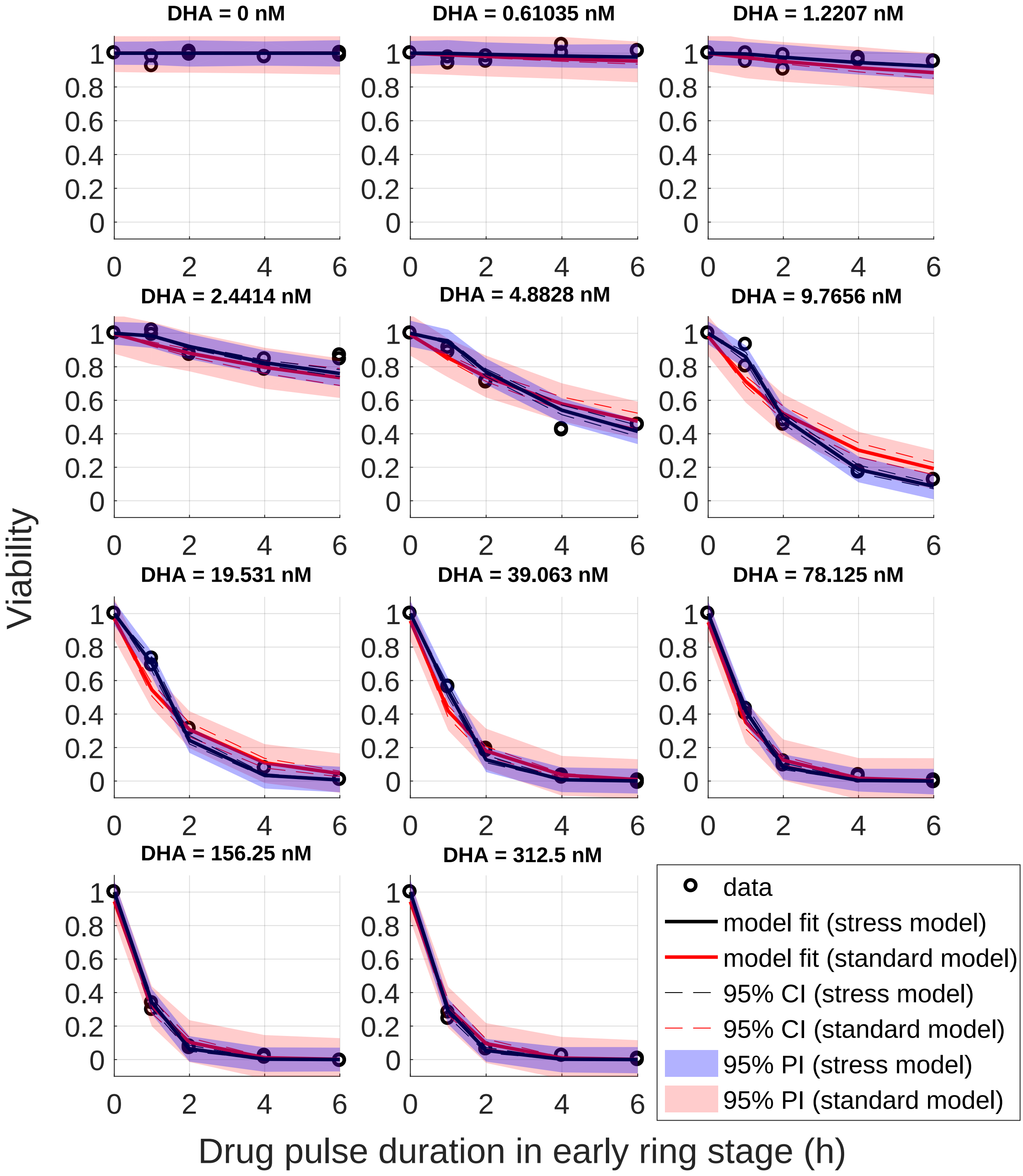

Figure S4: Comparison of the fitting results of the dynamic stress model to the standard model (early ring stage). The applied DHA concentration (which then decays) is indicated in the title of each panel. Empty circles are viability data points and duplicate data points for each condition are shown. Black curves show the predicted mean viability measurements from the dynamic stress model with fixed γ parameter. The best-fits with 95% confidence intervals (CI) and 95% prediction intervals (PI) are indicated in the legend. Note that the result for DHA = 625 nM are hidden by the legend but is very similar to that for DHA = 312.5 nM.

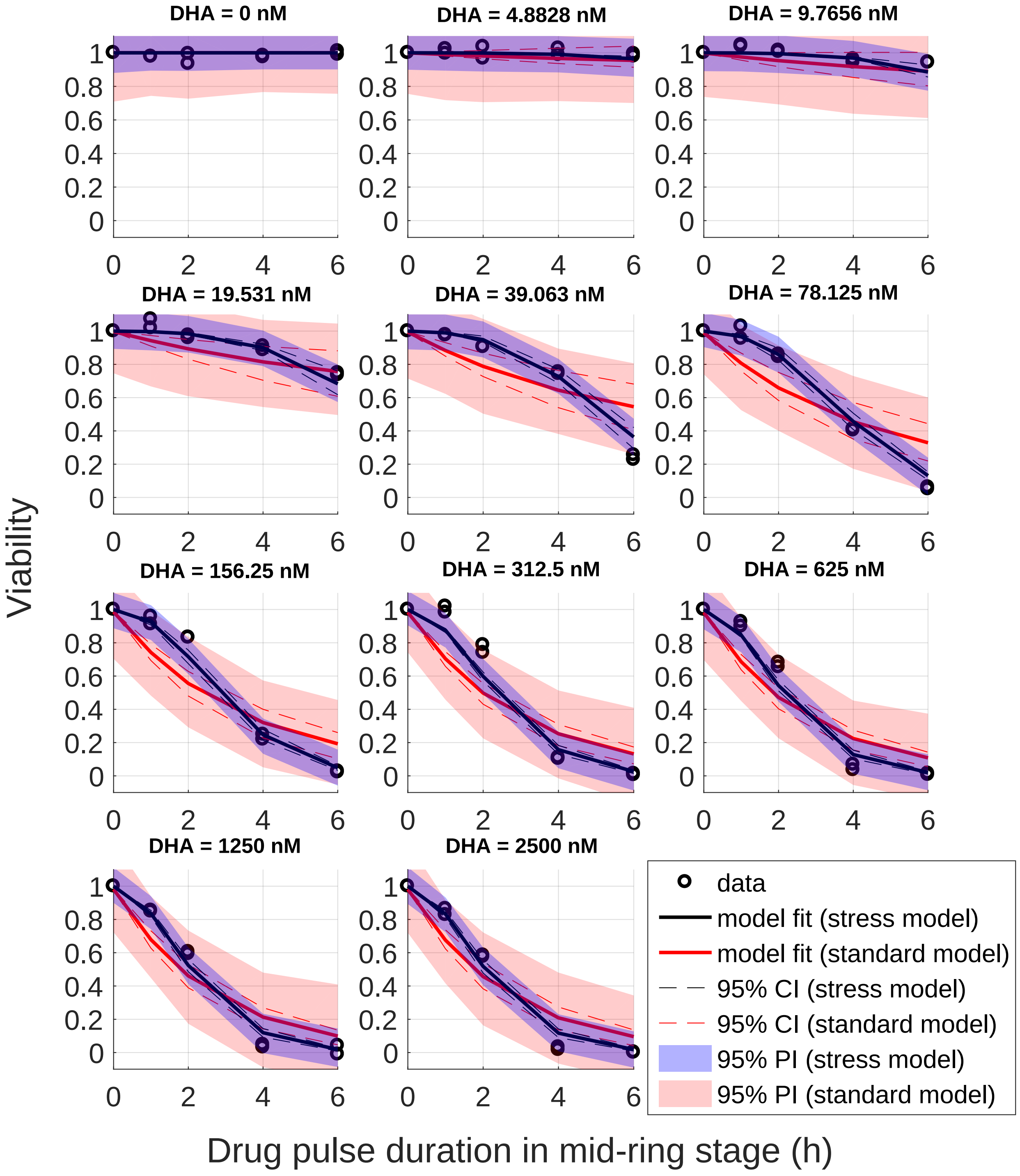

Figure S5: Comparison of the fitting results of the dynamic stress model to the standard model (mid-ring stage). The applied DHA concentration (which then decays) is indicated in the title of each panel. Empty circles are viability data points and duplicate data points for each condition are shown. Black curves show the predicted mean viability measurements from the dynamic stress model with fixed γ parameter. The best-fits with 95% confidence intervals (CI) and 95% prediction intervals (PI) are indicated in the legend. Note that the result for DHA = 5000 nM are hidden by the legend but is very similar to that for DHA = 2500 nM.

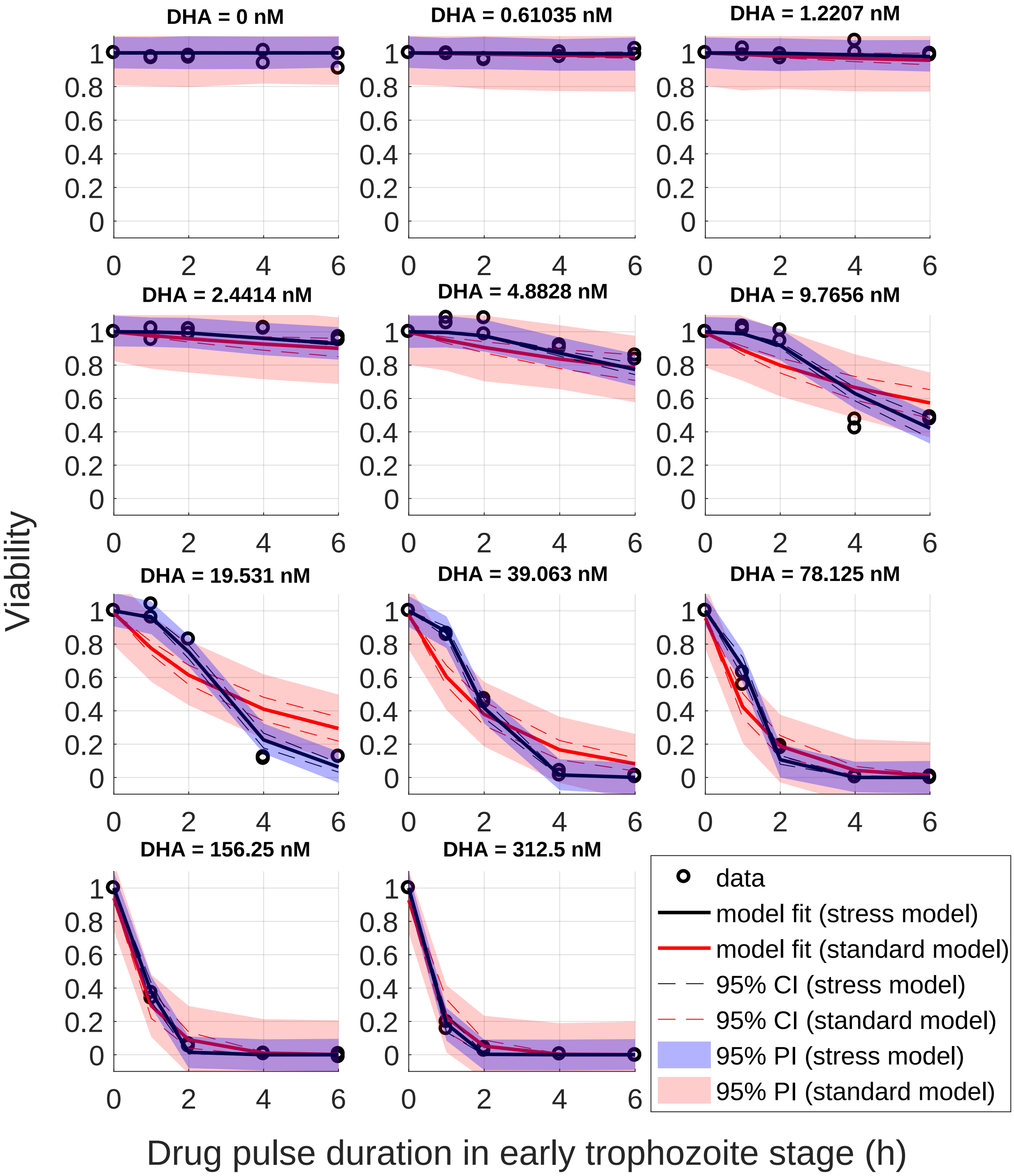

Figure S6: Comparison of the fitting results of the dynamic stress model to the standard model (early trophozoite stage). The applied DHA concentration (which then decays) is indicated in the title of each panel. Empty circles are viability data points and duplicate data points for each condition are shown. Black curves show the predicted mean viability measurements from the dynamic stress model with fixed γ parameter. The best-fits with 95% confidence intervals (CI) and 95% prediction intervals (PI) are indicated in the legend. Note that the result for DHA = 625 nM are hidden by the legend but is very similar to that for DHA = 312.5 nM.

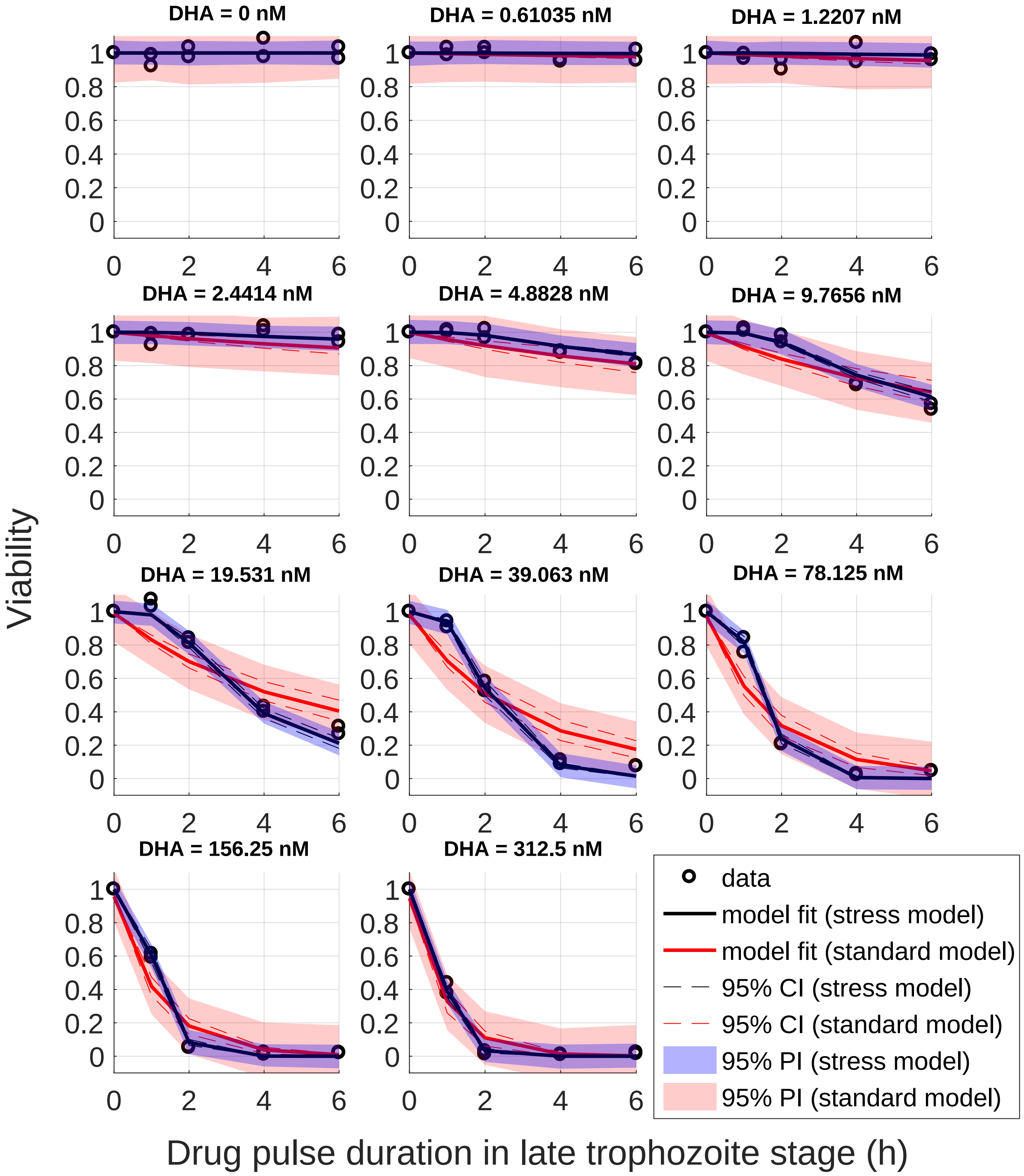

Figure S7: Comparison of the fitting results of the dynamic stress model to the standard model (late trophozoite stage). The applied DHA concentration (which then decays) is indicated in the title of each panel. Empty circles are viability data points and duplicate data points for each condition are shown. Black curves show the predicted mean viability measurements from the dynamic stress model with fixed γ parameter. The best-fits with 95% confidence intervals (CI) and 95% prediction intervals (PI) are indicated in the legend. Note that the result for DHA = 625 nM are hidden by the legend but is very similar to that for DHA = 312.5 nM.

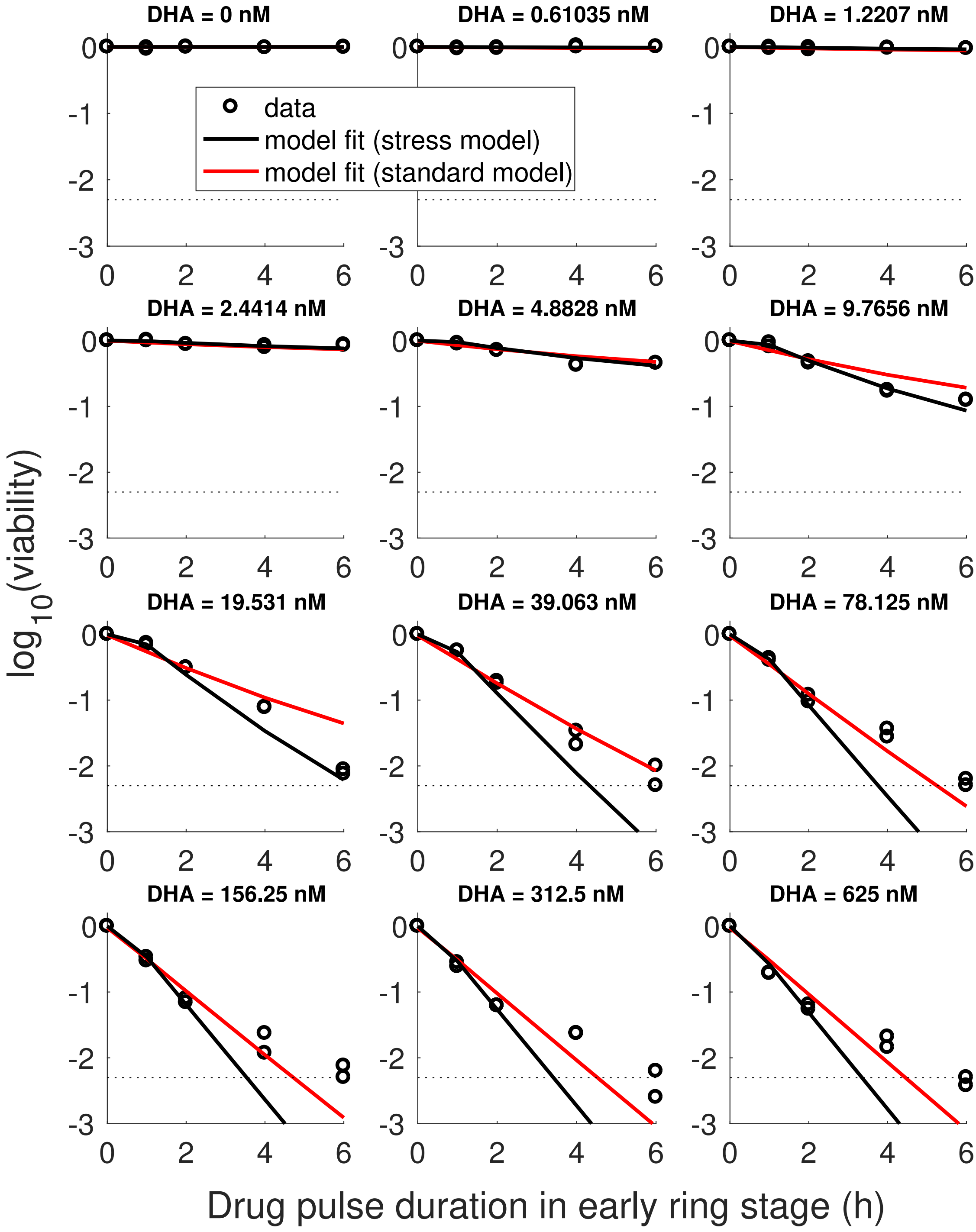

Figure S8: Comparison of the fitting results of the dynamic stress model to the standard model (early ring stage) in logarithm Y scale. The applied DHA concentration (which then decays) is indicated in the title of each panel. Empty circles are viability data points and duplicate data points for each condition are shown. Black curves show the fit by the dynamic stress model with fixed γ parameter and the red curves show the fit by the standard model. The dashed line is an estimated detection of limit (which is for indicative purpose and requires further experiments to refine).

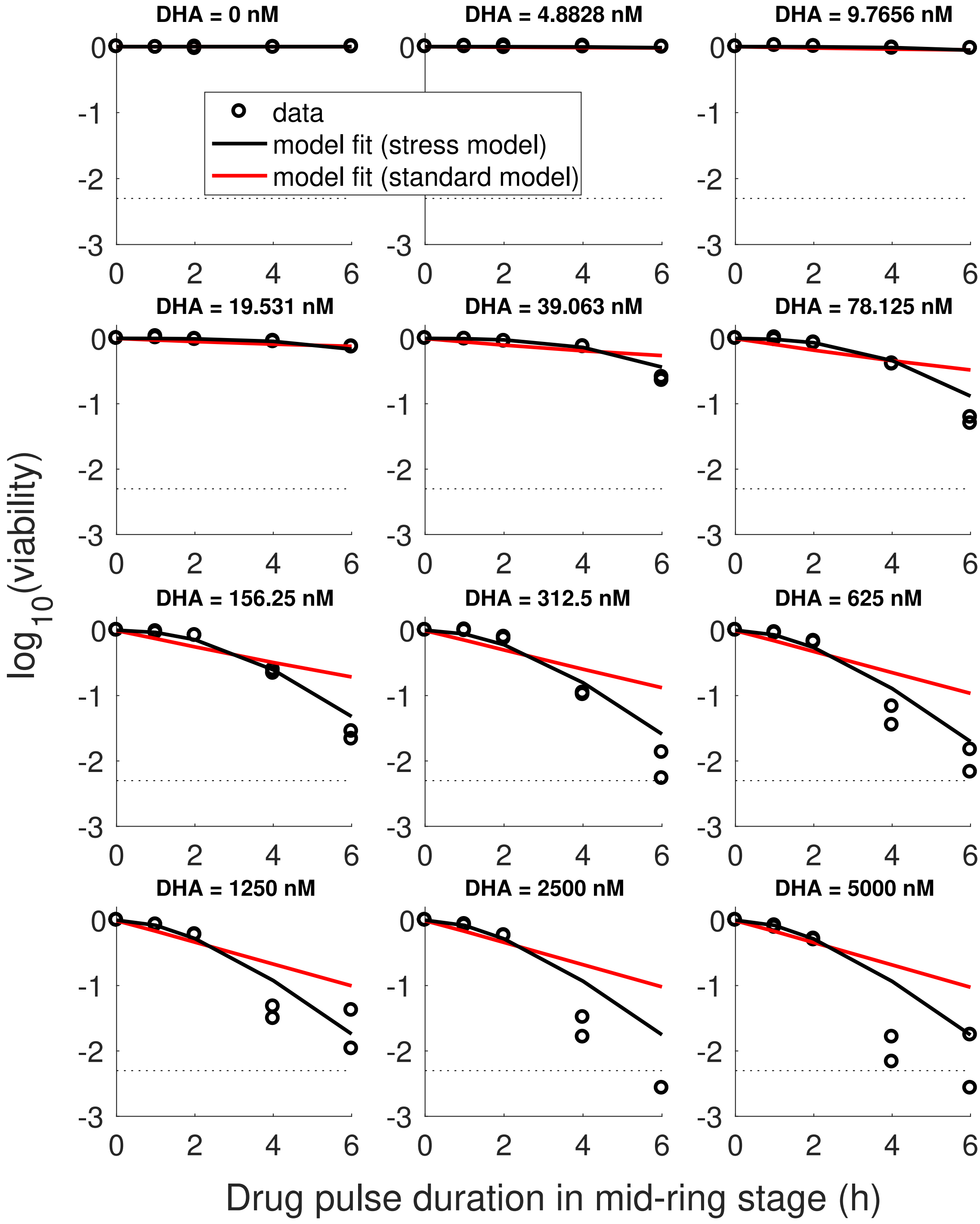

Figure S9: Comparison of the fitting results of the dynamic stress model to the standard model (mid-ring stage) in logarithm Y scale. The applied DHA concentration (which then decays) is indicated in the title of each panel. Empty circles are viability data points and duplicate data points for each condition are shown. Black curves show the fit by the dynamic stress model with fixed γ parameter and the red curves show the fit by the standard model. The dashed line is an estimated detection of limit (which is for indicative purpose and requires further experiments to refine).

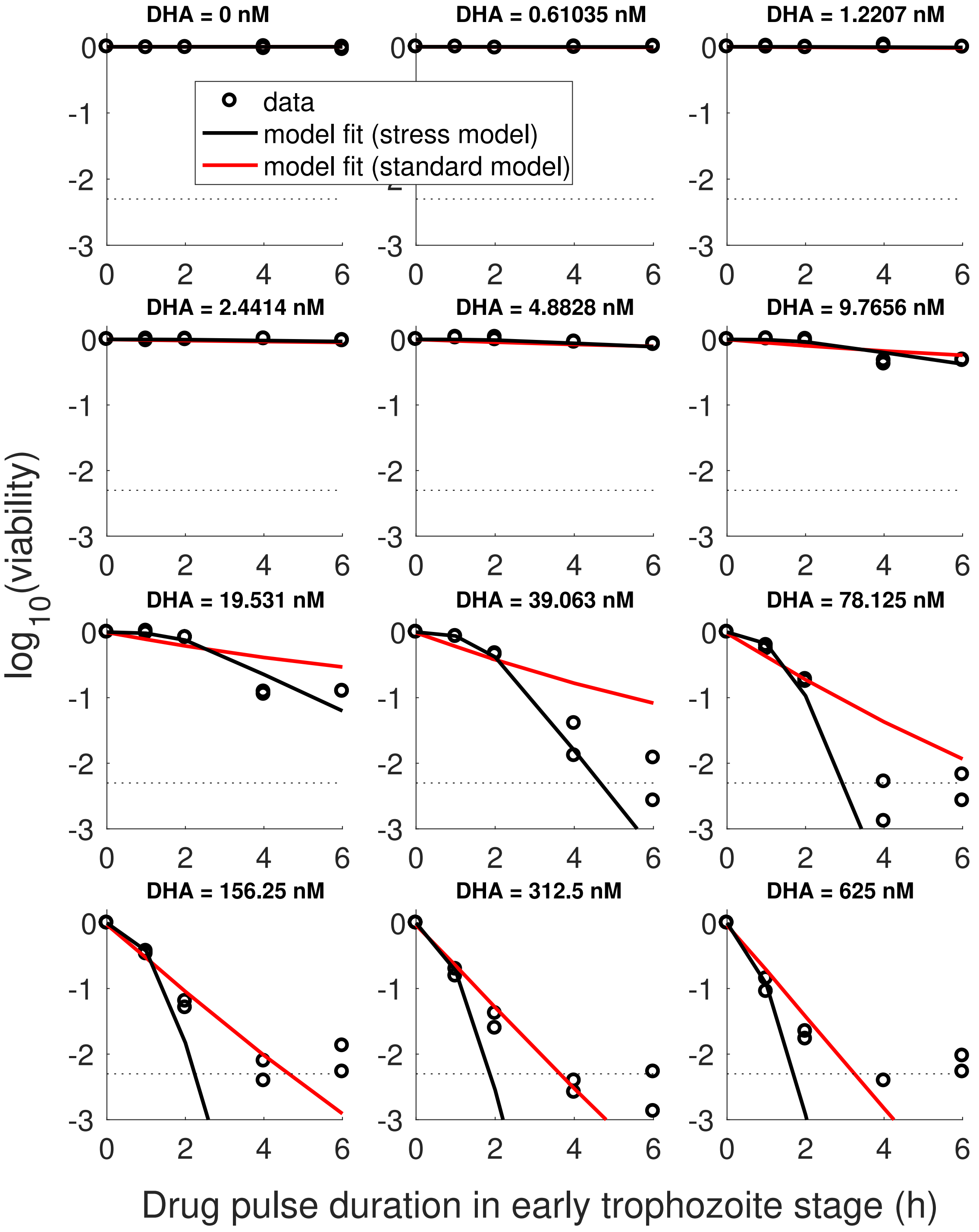

Figure S10: Comparison of the fitting results of the dynamic stress model to the standard model (early trophozoite stage) in logarithm Y scale. The applied DHA concentration (which then decays) is indicated in the title of each panel. Empty circles are viability data points and duplicate data points for each condition are shown. Black curves show the fit by the dynamic stress model with fixed γ parameter and the red curves show the fit by the standard model. The dashed line is an estimated detection of limit (which is for indicative purpose and requires further experiments to refine).

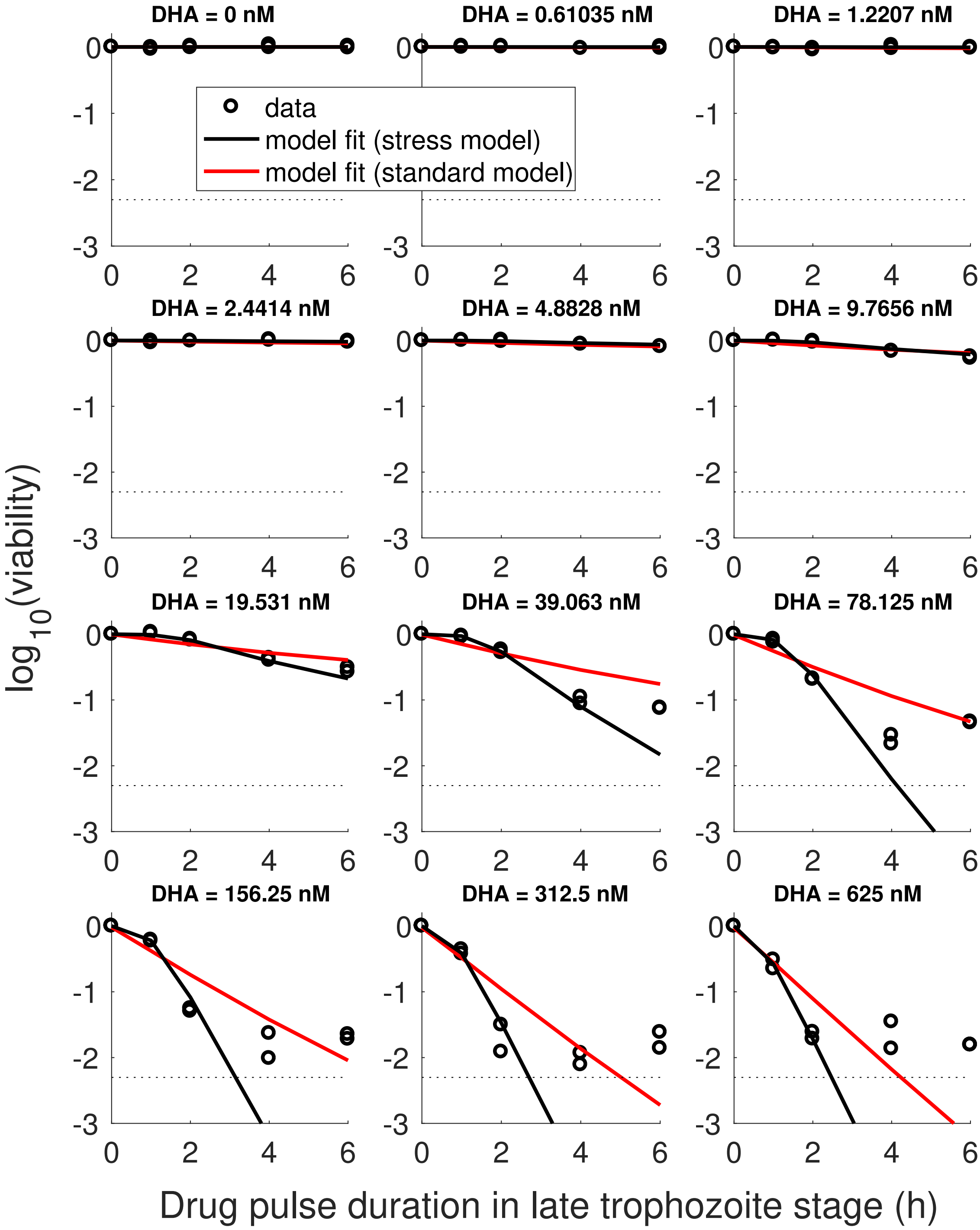

Figure S11: Comparison of the fitting results of the dynamic stress model to the standard model (late trophozoite stage) in logarithm Y scale. The applied DHA concentration (which then decays) is indicated in the title of each panel. Empty circles are viability data points and duplicate data points for each condition are shown. Black curves show the fit by the dynamic stress model with fixed γ parameter and the red curves show the fit by the standard model. The dashed line is an estimated detection of limit (which is for indicative purpose and requires further experiments to refine).

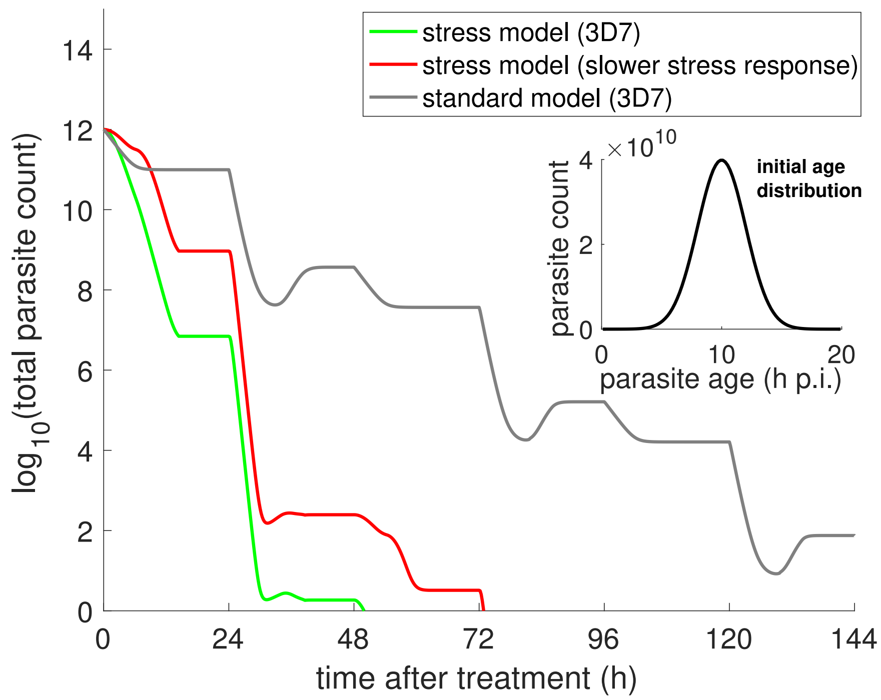

Figure S12: Comparison of *in vivo* simulation of parasite killing for the dynamic stress model and the standard model under a treatment of 2mg/kg artesunate every 24 hours. PK profile is a series of repeated DHA concentration profile every 24 hours (i.e. repeated simulations of DHA concentration profile in Fig.5A upper panel in the main text) and the first dose is applied at 0 h (the followings are at 24 h, 48 h, 72 h, ...). The green curve corresponding to the laboratory 3D7 strain is generated using the dynamic stress model and the parameters in Table1 (the same as that in Fig. 6), while the red curve is generated using the same model and the same set of parameters except for reducing λ for mid-ring stage to be 0.1 h^{-1} to simulate a more resistant strain (the same as that in Fig. 6). The grey curve corresponds is generated using the standard model and the parameters in Table S2. All other parameter values are provided in the legend of Fig. 6 in the main text.

Table S1: Results of fitting the model to viability data. The model-based 95% CI and parametric bootstrap 95% CI are introduced in *Materials and Methods* in the main text. γ is allowed to vary for different stages.

Parameter (unit)	Estimate	SE	Model-based 95% CI	Parametric bootstrap 95% CI
**Early ring stage**				
λ (h^{-1})	6.2913	0.5294	(5.2537, 7.3288)	(-0.2036, 9.5726)
γ	1.7703	0.1694	(1.4383, 2.1022)	(1.5789, 1.9794)
α (h^{-1})	1.6987	0.1484	(1.4078, 1.9896)	(1.0569, 1.8591)
β_1 (nM)	1013.0	361.39	(304.71, 1721.3)	(-76380, 1909.0)
β_2 (nM)	12.734	2.0654	(8.6858, 16.782)	(7.5398, 14.927)
**Mid-ring stage**				
λ (h^{-1})	0.3638	0.1359	(0.0965, 0.6301)	(0.2216, 0.5375)
γ	1.7433	0.2889	(1.1770, 2.3096)	(1.1612, 2.0665)
α (h^{-1})	1.1472	0.2806	(0.5973, 1.6971)	(0.3129, 1.4028)
β_1 (nM)	222.94	94.830	(37.076, 408.81)	(113.78, 302.00)
β_2 (nM)	9.86×10^{-4}	4.66×10^{-5}	$(8.90, 10.8) \times 10^{-4}$	$(9.70, 10.0) \times 10^{-4}$
**Early trophozoite stage**				
λ (h^{-1})	1.2720	0.2163	(0.8480, 1.6959)	(0.8361, 1.7458)
γ	2.0864	0.2023	(1.6900, 2.4829)	(1.6895, 2.3859)
α (h^{-1})	4.8326	0.6615	(3.5361, 6.1291)	(2.3239, 5.8950)
β_1 (nM)	280.55	72.477	(138.50, 422.60)	(60.544, 387.98)
β_2 (nM)	26.711	6.3515	(14.262, 39.160)	(13.965, 34.734)
**Late trophozoite stage**				
λ (h^{-1})	2.3076	0.3986	(1.5263, 3.0890)	(1.4744, 2.8986)
γ	1.5568	0.0799	(1.4001, 1.7134)	(1.4097, 1.6828)
α (h^{-1})	3.3892	0.3381	(2.7265, 4.0519)	(2.1815, 4.0184)
β_1 (nM)	1132.2	469.61	(211.78, 2052.6)	(-306.63, 1704.5)
β_2 (nM)	60.559	11.012	(38.976, 82.141)	(34.002, 74.003)

Comparison of the dynamic stress model to the standard model

Here we examine if the dynamic stress model (Eqs. 5-8 in the main text) is statistically superior to the standard killing rate model (Eq. 1 in the main text) when fitting to the *in vitro* viability data introduced in the main text. Note that the DHA concentration C is a function of time in both models and is given by Eq. 13 in the main text. Table S2 provides the parameter estimates and corresponding standard errors (SE) for the standard model for each parasite stage. The fitting results for the dynamic stress model have been provided in Table 1 in the main text. Table S3 provides the statistical comparison of the standard model and the dynamic stress model. For all parasite stages, the Akaike information criterion (AIC) indicate the dynamic stress model provides a better fit to the data than the standard model.

Table S2: Parameter estimates generated by fitting the standard model to the viability data for each parasite stage.

Parameters	early rings	mid-rings	early troph	late troph
k_{max}	1.20314	0.39521	1.78555	1.46733
K_c	18.4289	65.9683	79.4698	106.425
γ	1.37243	1.37605	1.23878	1.11382
σ_b	0.056766	0.136286	0.097876	0.080867
σ_w	0.021231	0.018298	0.025636	0.028777
SE of k_{max}	0.131559	0.05884	0.357089	0.289716
SE of K_c	5.48148	38.2638	35.3201	49.4705
SE of γ	0.184101	0.55469	0.148148	0.137433
SE of σ_b	0.000822	0.003338	0.002375	0.001659
SE of σ_w	0.000168	7.93E-05	0.000155	0.000179

Table S3: Akaike information criterion (AIC) comparing the standard model and the dynamic stress model. Each model was fitted separately to the viability data for each parasite stage. Improved AIC are indicated in yellow. Note that the parameters include both the model parameters in the killing rate equations and two parameters related to the residue error (i.e. σ_b and σ_w).

Model	Number of model parameters	OFV[a]	AIC[b]
**early ring**			
standard model	5	-597.786	-587.786
dynamic stress model	7	-663.7349334	-649.735
**mid-ring**			
standard model	5	-522.743	-512.743
dynamic stress model	7	-632.9048535	-618.905
**early troph**			
standard model	5	-470.86	-460.860
dynamic stress model	7	-567.0569428	-553.057
**late troph**			
standard model	5	-547.987	-537.987
dynamic stress model	7	-683.9687299	-669.969

[a]OFV = objective function value = -2×ln(L), where ln(L) is the log-likelihood for the model.

[b]AIC = Akaike information criterion = 2×p – 2×ln(L), where p is the number of model parameters and ln(L) is the log-likelihood for the model.
